# Odd-Number Cyclo[*n*]Carbons Sustaining Alternating Aromaticity

**DOI:** 10.1021/acs.jpca.1c08507

**Published:** 2022-04-14

**Authors:** Glib V. Baryshnikov, Rashid R. Valiev, Lenara I. Valiulina, Alexandr E. Kurtsevich, Theo Kurtén, Dage Sundholm, Michael Pittelkow, Jinglai Zhang, Hans Ågren

**Affiliations:** † College of Chemistry and Chemical Engineering, Henan University, Kaifeng 475004, Henan, P. R. China; ‡ Laboratory of Organic Electronics, Department of Science and Technology, Linköping University, Norrköping SE-60174, Sweden; § Department of Chemistry, Faculty of Science, University of Helsinki, FIN-00014 Helsinki, Finland; ∥ Department of Optics and Spectroscopy, Tomsk State University, Tomsk 634050, Russia; ⊥ Department of Chemistry, University of Copenhagen, Copenhagen Ø DK-2100, Denmark; # Department of Physics and Astronomy, Uppsala University, Uppsala SE-75120, Sweden

## Abstract

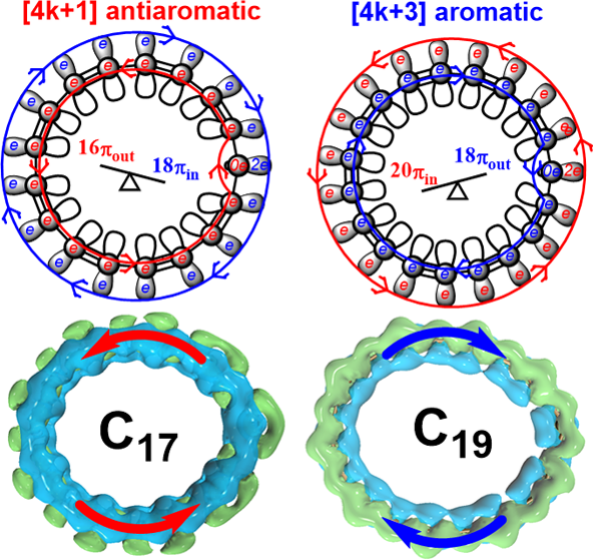

Cyclo[*n*]carbons (*n* = 5, 7, 9, ..., 29) composed from an odd number of carbon atoms are studied computationally at density functional theory (DFT) and *ab initio* complete active space self-consistent field (CASSCF) levels of theory to get insight into their electronic structure and aromaticity. DFT calculations predict a strongly delocalized carbene structure of the cyclo[*n*]carbons and an aromatic character for all of them. In contrast, calculations at the CASSCF level yield geometrically bent and electronically localized carbene structures leading to an alternating double aromaticity of the odd-number cyclo[*n*]carbons. CASSCF calculations yield a singlet electronic ground state for the studied cyclo[*n*]carbons except for C_25_, whereas at the DFT level the energy difference between the lowest singlet and triplet states depends on the employed functional. The BHandHLYP functional predicts a triplet ground state of the larger odd-number cyclo[*n*]carbons starting from *n* = 13. Current-density calculations at the BHandHLYP level using the CASSCF-optimized molecular structures show that there is a through-space delocalization in the cyclo[*n*]carbons. The current density avoids the carbene carbon atom, leading to an alternating double aromaticity of the odd-number cyclo[*n*]carbons satisfying the antiaromatic [4k+1] and aromatic [4k+3] rules. C_11_, C_15_, and C_19_ are aromatic and can be prioritized in future synthesis. We predict a bond-shift phenomenon for the triplet state of the cyclo[*n*]carbons leading to resonance structures that have different reactivity toward dimerization.

## I Introduction

**Figure 1:**
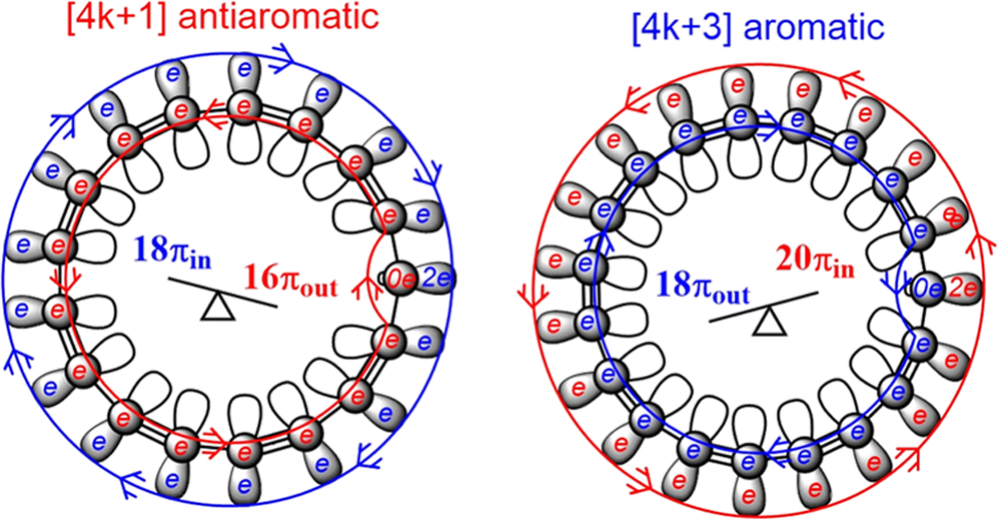
Mixed aromatic and antiaromatic character of C_17_ (left) as compared to the one for C_19_ (right). The net antiaromatic C_17_ is π antiaromatic and σ aromatic and vice versa for the net aromatic C_19_. Red and blue arrows denote the paratropic and diatropic currents, respectively; π_in_ and π_out_ mean that the electrons are aligned in molecular plane and perpendicular to it, respectively.

Cyclo[*n*]carbons represent a unique and underexplored form of carbon molecules among numerous other allotropes.^1^ They have been observed in gas-phase mass-spectra experiments.^2−6^ The first ever purposeful chemical synthesis of an even-number cyclo[18]carbon (C_18_) was carried out by Kaiser et al. in 2019,^7^ while last year, Scriven et al. improved the synthetic protocol for cyclo[18]carbon, reaching a reaction yield of 64%.^8^ After these successful synthesis endeavors, a large number of articles have been devoted to studies of the electronic structure of cyclo[*n*]carbons. Most of them focus on the even-number members of this class of molecules.^9−21^ Studies on odd-number cyclo[*n*]carbons have been limited to very few articles that mainly report properties of small carbon rings up to C_17_ calculated at the DFT level of theory.^22−26^ These articles were mainly aimed to describe the relative stability of linear vs cyclic isomers. The aromaticity and electronic properties of odd-number cyclo[*n*]carbons were studied for the first time in 2009 by Fowler et al. at the B3LYP/6-31G(d) level of theory.^27^ They concluded that [4k+1] and [4k+3] cyclo[*n*]carbons contain [4k+2] electrons in the out-of-plane π-system, while the residual [4k] or [4k+1] electrons belong to the in-plane π-system. As a result, they found that all odd-number cyclo[*n*]carbons sustain a diatropic ring current in the out-of-plane π-system, which is stronger than the paratropic ring current in the in-plane π-system, i.e., they found that the studied odd-number cyclo[*n*]carbons (C_7_–C_29_) are globally aromatic and called them “π aromatic and σ antiaromatic”.^27^ Here, we challenge this conclusion since the employed DFT calculations are not able to properly describe the carbene structure of the odd-number cyclo[*n*]carbons. Recently, Seenithurai and Chai^28^ systematically studied odd-number cyclo[*n*]carbons containing up to 99 carbon atoms at the TAO-DFT-LDA level of theory and showed that some electronic descriptors like the ionization potential, electron affinity, and the optical gap alternate with *n*. They did not obtain any clear oscillations in the singlet–triplet energy gap (Δ*E*_ST_) but reported Δ*E*_ST_ of 4–5.6 kcal mol^–1^ for C_13_–C_49_, which is remarkable when considering the polyradical nature of these species. As Seenithurai and Chai^28^ unfortunately did not provide any analysis of the optimized molecular structures of the studied odd-number cyclo[*n*]carbons, we cannot judge how well their structures agree with the ones we report here. Hobza et al.^29^ predicted very recently a triplet ground state for C_17_ based on ωB97XD/def2-TZVPP calculations, which is most likely incorrect due to the limitations of the employed DFT level to describe the localized carbene structure of odd-number cyclo[*n*]carbons. Here, we report and analyze the electronic and molecular structures of odd-number cyclo[*n*]carbons obtained at the CASSCF level of theory. Current densities are calculated at the DFT level using the BHandHLYP functional to understand the electronic structure properties and the aromatic character of cyclo[*n*]carbons (*n* = 5, 7, 9, ..., 29). We utilize the gauge-including magnetically induced currents (GIMIC) method^30,31^ for analyzing the aromatic properties. Based on these calculations, we propose a novel concept of through-space aromaticity of the odd-number cyclo[*n*]carbons in the singlet ground state, which is schematically presented in Figure 1. The calculations show that the electron delocalization avoids the empty p_out_ orbital at the carbene atom, leading to alternating aromaticity of the odd-number cyclo[*n*]carbons. The cyclo[*n*]carbons with [4k+1] carbon atoms are antiaromatic, whereas those with [4k+3] carbon atoms are aromatic, where k is a positive integer.

## II Computational Details

The molecular structures of the lowest singlet and triplet states of cyclo[2*n*+1]carbons (*n* = 2–14) were initially optimized at the DFT level using the hybrid functional BHandHLYP^32,33^ and the Karlsruhe def2-TZVP basis set.^34^ Since previous studies of the electronic structure of even-numbered cyclo[*n*]carbons showed that the experimentally observed polyyne structures are obtained only when using a functional with a large amount of the Hartree–Fock exchange (>40%),^10,15^ we used here the BHandHLYP functional that satisfies this condition with 50% of the Hartree–Fock exchange. We also optimized the molecular structures using functionals with different amounts of Hartree–Fock exchange: TPSSh (0%),^35^ B3LYP (20%),^33,36^ BMK (42%),^37^ and M06HF (100%).^38^

The molecular structures of the lowest singlet and triplet states were also optimized at the *ab initio* complete active space self-consistent field (CASSCF) level,^39,40^ since multiconfiguration SCF calculations yielded the experimentally observed polyyne structure for the even-numbered cyclo[*n*]carbons.^10^ The 6-31G(d,p)^41,42^ basis set was used in the optimization of the molecular structures at the CASSCF level. We used the (14,12) active space (14 electrons in 12 active orbitals) for all of the studied molecules. Smaller and larger active spaces were also used for checking the choice of the active space. The structure optimizations at the CASSCF level were performed using the Firefly software.^43,44^ We also performed structure optimizations of the lowest singlet and triplet states of C_11_ at the multireference second-order perturbation theory (CASPT2) level using split-valence polarization basis sets (SVP)^45,46^ with the BAGEL program.^47^

Calculations of the magnetically induced current densities were carried out using the gauge-including magnetically induced currents (GIMIC) method.^48,49^ The basis-set information, the atomic orbital density matrix, and the perturbed atomic orbital density matrices are the input data of the GIMIC calculations. The density matrices are obtained by performing NMR shielding calculations.

The NMR shielding calculations were performed at the BHandHLYP/def2-TZVP level using the BHandHLYP and CASSCF structures. The first-order magnetically perturbed density matrices and the DFT-optimized molecular structures of the cyclo[2*n*+1]carbons (*n* = 2–14) were computed using the Gaussian 16 program package.^50^ A script was used for converting the Gaussian output to GIMIC input data in appropriate format.^48,49^ The strengths of the magnetically induced ring currents (*I* in nA T^–1^) were obtained by integrating the current-density flux that passes through a plane placed perpendicular to the molecular plane. The visualization of the current densities was performed within the Multiwfn software.^51^ We also calculated NMR shielding tensors at CASSCF/SVP^45,46^ level of theory for singlet and triplet states of the C_11_ and C_13_ cyclocarbons using DALTON software.^52^ The ring-current strengths were then obtained at the CASSCF level by integrating the *zz* component of the shielding tensor along the symmetry axis in the middle of the ring using the Ampère–Maxwell’s law.^53^

## III Results and Discussion

### III.I Electronic Structure of Even-Number Cyclocarbons

The even-number cyclo[*n*]carbons are doubly aromatic according to the Hückel rules for antiaromatic [4k] and aromatic [4k+2] rings with *k* = 1–8.^9^ The aromaticity is maintained by the delocalized in-plane (p_in_) and out-of-plane (p_out_) orbitals, respectively,^9^ which can also be interpreted as double σ (in-plane) and π (out-of-plane) aromaticity and antiaromaticity of the [4k+2] and [4k] species. We recently showed that doubly aromatic C_18_ sustains a stronger magnetically induced ring current (MIRC) in the 18π_out_ out-of-plane electrons (*I*_out_ = 21.8 nA T^–1^) than in the 18π_in_ in-the-plane electrons (*I*_in_ = 7.2 nA T^–1^) due to the perpendicular orientation of the p_out_ atomic orbitals (AO) with respect to the magnetic field direction. The radial p_in_ AOs have an external CCC angle of 200° affecting the electron delocalization.^10^ For *k* > 8, the even-number cyclo[*n*]carbons have a pronounced bond alternation with a transition to nonaromaticity occurring for the [4k] and [4k+2] species.^9^ The larger even-number cyclo[*n*]carbons do not sustain any MIRC. The results of the GIMIC calculations agree completely with the results obtained with simple independent-particle simulations by Bylaska et al.^54^

### III.II Electronic Structure of Odd-Number Cyclocarbons

The odd-number cyclo[*n*]carbons have an extra carbon atom (a carbene) affecting their electronic structure. It is well known that carbenes generally have a triplet ground state with a nonlinear molecular geometry.^55,56^ Thus, a triplet ground state can be anticipated also for odd-number cyclo[*n*]carbons, and indeed the BHandHLYP/def2-TZVP calculations suggest that C_13_ and larger odd-number cyclo[*n*]carbons have a triplet ground state. For C_13_, the triplet state is 8.5 kcal mol^–1^ below the singlet state. The energy difference between the singlet and triplet states (Δ*E*_ST_) increases for larger rings and reaches 21.2 kcal mol^–1^ for C_29_ (see Table 1). Calculations on C_17_ employing DFT functionals with different amounts of Hartree–Fock exchange (from 0 to 100%) do not qualitatively change the trend; see Table S1.

**Table 1:** Energy Difference (Δ*E*_ST_, kcal mol^–1^) between the Lowest Singlet (S) and Triplet (T) States and the Strength of the Magnetically Induced Ring Current (MIRC) (*I*, nA T^–1^) for the S and T States Calculated at the BHandHLYP/def2-TZVP and CASSCF(14;12)/SVP Levels of Theory^a^

	BHandHLYP/def2-TZVP			CASSCF(14;12)		
	Δ*E*_ST_	*I*(S)	*I*(T)	Δ*E*_ST_	*I*(S)^b^^,^^d^	*I*(T)^b^^,^^d^
C_5_	–2.0	14.3	4.8	–16.0	15.0/16.4	8.2/3.3
C_7_	–16.8	14.3	1.4	–20.3	13.2/13.4	16.4/17
C_9_	–1.3	–0.8	–13.9	–8.8	–32.8/–45.2	–9.1/–16.5
C_11_	–5.2	14.9	–2.0	–40.2	12.0/12.3	0.9/2.2
					12.0^c^	5.2^c^
C_13_	8.5	2.7	–6.7	–5.6	–20.7/–27.0	–5.7/–7.3
					–9.0^c^	–1.5^c^
C_15_	5.3	12.1	–1.1	–27.3	9.0/9.0	0.6/0.7
C_17_	15.1	3.2	–2.1	–1.4	–10.9/–12.8	–3.2/–2.9
C_19_	12.5	8.4	–1.1	–25.4	5.5/5.5	0.1/0.2
C_21_	19.2	3.8	–0.1	–0.1	–5.9/–5.7	–1.5/–3.0
C_23_	17.0	5.2	–0.9	–24.2	3.0/1.7	0.3/–1.4
C_25_	19.4	5.8	–0.5	0.8	–3.2/–1.8	–0.9/1.0
C_27_	19.9	3.0	–0.6	–15.2	1.4/0.8	–0.1/–2.0
C_29_	21.3	2.8	0.6	–1.9	–1.8/–0.7	–0.6/2.1

a The MIRC has also been calculated at other levels of theory.

b I(S) and I(T) calculated at the BHandHLYP/6-31G(d,p) level using the CASSCF-optimized geometries.

c I(S) and I(T) calculated at the CASSCF/SVP level using the CASSCF-optimized geometries.

d I(S) and I(T) calculated at the M06-2X/6-31G(d,p) level using the CASSCF-optimized geometries.

However, calculations at the CASSCF level with an active space consisting of 14 electrons in 12 orbitals predict that most of the studied cyclo[*n*]carbons have a singlet ground state, since Δ*E*_ST_ is positive for the [4k+1] and [4k+3] molecules, except for C_25_ whose singlet and triplet states are nearly degenerate. Calculations with different sizes of the active space do not qualitatively change the picture for the [4k+3] molecules; see Table S1. For the antiaromatic [4k+1] cyclo[*n*]carbons (*k* = 1–7), Δ*E*_ST_ decreases faster than for the aromatic [4k+3] ones as seen in Table 1. The [4k+1] rings of C_21_, C_25_, and C_29_ (*k* = 5, 6, and 7, respectively) are characterized by a quasi-degenerate singlet–triplet ground state, while for the [4k+3] systems (*k* = 2–6), Δ*E*_ST_ is large and negative with values between −40 kcal mol^–1^ for C_11_ and −15 kcal mol^–1^ for C_27_.

The CASSCF calculations yield a low-lying singlet ground state for the [4k+3] species due to aromatic stabilization. The smaller rings sustain a strong diatropic MIRC (*I*(S) in Table 1). The singlet–triplet splitting is small for the [4k+1] cyclo[*n*]carbons, which are antiaromatic, sustaining a paratropic MIRC in the lowest singlet state. Calculations of the [4k+1] species with an active space that is larger than 14 electrons in 12 orbitals may change the order of the singlet and the triplet states for the larger rings. However, such CASSCF calculations are computationally expensive. DFT calculations at the BHandHLYP/def2-TZVP level that work perfectly for the even-number cyclo[*n*]carbons^9^ yield a triplet ground state for larger odd-number cyclo[*n*]carbons starting from C_11_. However, single-point calculations using the CASSCF molecular structures and the B3LYP,^33,36^ O3LYP,^33,57^ M06-2X,^58^ and ωB97XD^59^ functionals qualitatively yield the same trend for Δ*E*_ST_ as obtained at the CASSCF level (see Table S2). The calculations thus show that different trends for Δ*E*_ST_ are obtained when using the DFT-optimized structures and the CASSCF ones.

Despite the fact that BHandHLYP and M06-2X functionals give quantitatively different values for the Δ*E*_ST_ gap (Table S2) when using CASSCF geometries, the strength of the MIRC for the S and T states calculated with these functional is largely the same (Table 1), implying that the strength of the MIRC values is almost independent of the employed level of DFT theory, while it strongly depends on the employed molecular structure. There is only a qualitative correlation between the Δ*E*_ST_ gap and the MIRC strength. An alternating S–T gap leads to a similar alternation in the aromatic/antiaromatic behavior of the odd-number cyclocarbons (Tables 1 and S2).

### III.III Structural Features of Odd-Number Cyclocarbons

**Figure 2:**
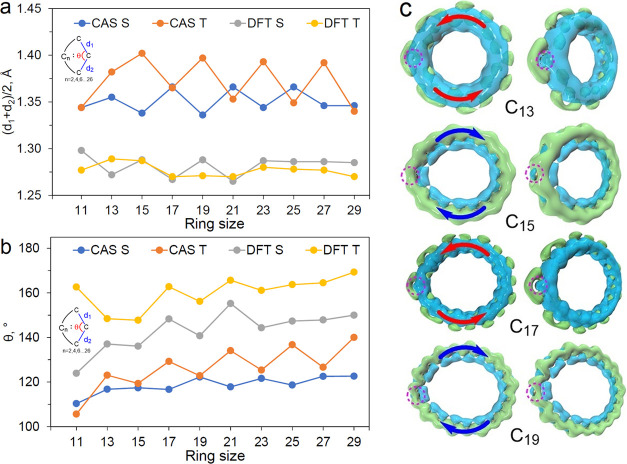
Average C–C distance (a) and the bond angle (b) at the carbene atom in cyclo[*n*]carbons C_11_–C_29_ calculated at the DFT and CASSCF levels of theory. Magnetically induced current density (c) of the singlet state of C_13_–C_19_ calculated at the BHandHLYP/6-31G(d,p) level using the CASSCF-optimized structures. The dashed circles indicate the hole corresponding to the empty p_out_ orbital. The green and blue isosurfaces denote the diatropic and paratropic current densities, respectively.

The molecular structures optimized at the BHandHLYP and CASSCF levels are shown in Table S3. The optimized structures at the two levels of theory are very different even though both levels predict a polyyne-type structure with alternating CC bonds. The BHandHLYP/6-31G(d,p) calculations yield a strongly delocalized carbene structure for the singlet (S) and triplet (T) states, i.e., the lone-pair electrons are not localized to a single carbon atom, resulting in short CC bonds (*d*_1_ and *d*_2_) near the carbene center and an obtuse CCC angle (θ) (Figure 2). Similar structural trends are obtained for the θ angle and the *d*_1_ and *d*_2_ bond lengths of the singlet and triplet states with increasing ring size. The strong lone-pair delocalization and the bond-length equalization obtained at the DFT level lead to a triplet ground state for most of the studied odd-number cyclo[*n*]carbons starting from C_13_.

The molecular structures of the singlet and triplet states of C_11_–C_29_ optimized at the CASSCF level have a sharp θ angle at the carbene atom. θ is in the range of 110–123° for the singlet states and 105–140° for the triplet states. The average CC bond lengths at the carbene atom are considerably longer for the CASSCF-optimized molecular structures as compared to the DFT ones (Figure 2). A clear alternation of the structural parameters θ, *d*_1_, and *d*_2_ is obtained for singlet [4k+1] and [4k+3] cyclo[*n*]carbons, suggesting that they have an alternating antiaromatic and aromatic character depending on the number of carbon atoms. The [4k+1] rings have small θ angles and long *d*_1_/*d*_2_ distances that result in an isolation of the carbene atom. The [4k+3] rings show the opposite trend—they have a large θ angle and short *d*_1_/*d*_2_ distances, which implies that the electrons of the carbene atom participate in electron delocalization. The θ and (*d*_1_ + *d*_2_)/2 structural parameters for the triplet state have the opposite trend as compared to the singlet state because the p_in_ and p_out_ orbitals are singly occupied. In the triplet state, the electron repulsion is weaker between the p_in_ orbitals but stronger between the p_out_ orbitals than for the singlet state. The singlet states have the p_in_ and p_out_ orbitals formally doubly occupied and nonoccupied, respectively.

### III.IV Aromaticity of Odd-Number Cyclocarbons

The structural features of the odd-number cyclo[*n*]carbons and particularly the θ angle as well as the *d*_1_ and *d*_2_ bond lengths of the carbene moiety determine their aromatic character. Since the lone-pair electron is delocalized at the BHandHLYP/def2-TZVP level, the singlet state of the studied odd-number cyclo[*n*]carbons is aromatic and sustains a net diatropic ring current according to the double aromaticity nature proposed by Fowler et al.^27^ The triplet state, which is the ground state for C_13_ and larger odd-number cyclo[*n*]carbons at the BHandHLYP/def2-TZVP level, is nonaromatic or weakly antiaromatic (Table 1). However, the triplet nature of the ground state is an artifact of the employed DFT method, while CASSCF calculations predict a low-lying aromatic singlet state of the [4k+3] cyclo[*n*]carbons and quasi-degenerate singlet and triplet ground state for the antiaromatic [4k+1] ones. The global electron delocalization avoids the carbene atom of the [4k+1] cyclo[*n*]carbons affecting their aromaticity. The aromatic pathway consists of the [4k] π_out_ electrons resulting in a global antiaromatic character of the [4k+1] rings (C_9_, C_13_, C_17_ etc.), while the participation of the π_in_ electrons in the global delocalization is prevented by the small θ angle. This is clearly seen in the magnetically induced ring currents shown in Figure 2c and Table S4. The formally empty p_out_ orbital of the [4k+3] cyclo[*n*]carbons is partially occupied due to interactions with the neighboring p_out_ orbitals. In this case, the carbene atom is less isolated because θ is larger and *d*_1_/*d*_2_ are shorter than for the [4k+1] rings. Thus, the [4k+2] π_out_ electrons contribute to the global diatropic out-of-plane current and a weaker paratropic ring current inside the ring (Figure 2c). The [4k+3] rings (C_11_, C_15_, C_19_, etc.) are therefore globally aromatic with a low-lying singlet ground state. Since the [4k+3] rings are the most stable molecules among the studied ones, they could be the main target for future synthesis attempts.

### III.V CASSCF and CASPT2 Calculations for C_11_ and C_13_

Similar to the case of even-number cyclocarbons,^9^ the CASSCF/6-31G(d,p) optimization for all the studied cyclocarbons predicts a significant contribution by only one main determinant into the wave function of the singlet and triplet states. The weights of the [2222222.....] and [22222211....] determinants are close to 0.9 for singlet and triplet states, respectively. Here “2” means doubly occupied orbital, “1” is a singly occupied orbital and “.” (dot) is an unoccupied orbital. Structure optimization of the C_11_ molecule in singlet and triplet states at CASPT2/SVP level provides similar orbital occupations as the CASSCF/6-31G(d,p) calculation. The weights of the [2222222.....] and [22222211....] determinants are 0.877 and 0.899, respectively, while the other determinants like [22222.2.2...] for singlet state and [22222.112...] for triplet state contribute with weights of about 0.1. Thus, the nondynamic (static) correlation effects for odd-number cyclocarbons are small, but could nevertheless be important. In our previous study on even-numbered cyclocarbons, we showed that static correlation effects decrease with increasing ring size. For C_11_, our CASPT2 calculations predict a negative Δ*E*_ST_ of −4.7 kcal mol^–1^ as also obtained at the CASSCF level. However, the CASPT2 value is significantly smaller than the one of −40.2 kcal mol^–1^ obtained at the CASSCF/6-31G(d,p) level. The magnetically induced ring currents of *I*(S) = 30 nA T^–1^ and *I*(T) = −1.0 nA T^–1^ calculated at BHandHLYP/def2-TZVP level using CASPT2/SVP-optimized geometries qualitatively agree with the ones of *I*(S) = 12 nA T^–1^ and *I*(T) = 0.9 nA T^–1^ obtained when using CASSCF/6-31G(d,p)-optimized geometries. The stronger ring current for the singlet state of the CASPT2/SVP-optimized C_11_ structure originates from the much smaller θ angle of only 70° between the *d*_1_ and *d*_2_ bonds at the carbene atom as compared to 110° for CASSCF/6-31G(d,p) geometry. Thus, the two atoms next to the carbene atom are much closer in the CASPT2/SVP structure (1.589 Å) than in the CASSCF/6-31G(d,p) geometry (2.204 Å). The CASPT2 calculation confirms the idea that avoiding the empty p_out_ orbital on the carbene carbon atom results in a [4*n*+2] aromatic π_out_ system, leading to a global aromaticity of the C_11_ molecule in the singlet ground state.

To exclude eventual errors related to the single-reference picture of the electronic structure provided by DFT, we performed calculations of the magnetic shielding tensors for the singlet and triplet states of the C_11_ and C_13_ compounds at the CASSCF level. Integrating the *zz* component of the magnetic shielding tensor along the symmetry axis in the middle of the ring yields the strength of the magnetically induced ring current according to Ampère–Maxwell’s law.^53^ The ring-current strength calculated at the BHandHLYP and CASSCF levels for the singlet state of C_11_ agree, whereas the CASSCF calculation suggests a stronger ring current for the triplet state than that obtained at the BHandHLYP level. Both levels of theory suggest that the singlet and triplet states of C_13_ are antiaromatic. The ring-current strengths calculated at the BHandHLYP level are 2–4 times larger that the CASSCF values (Table 1).

### III.VI Reactivity of Triplet-State Odd-Number Cyclocarbons

**Figure 3:**
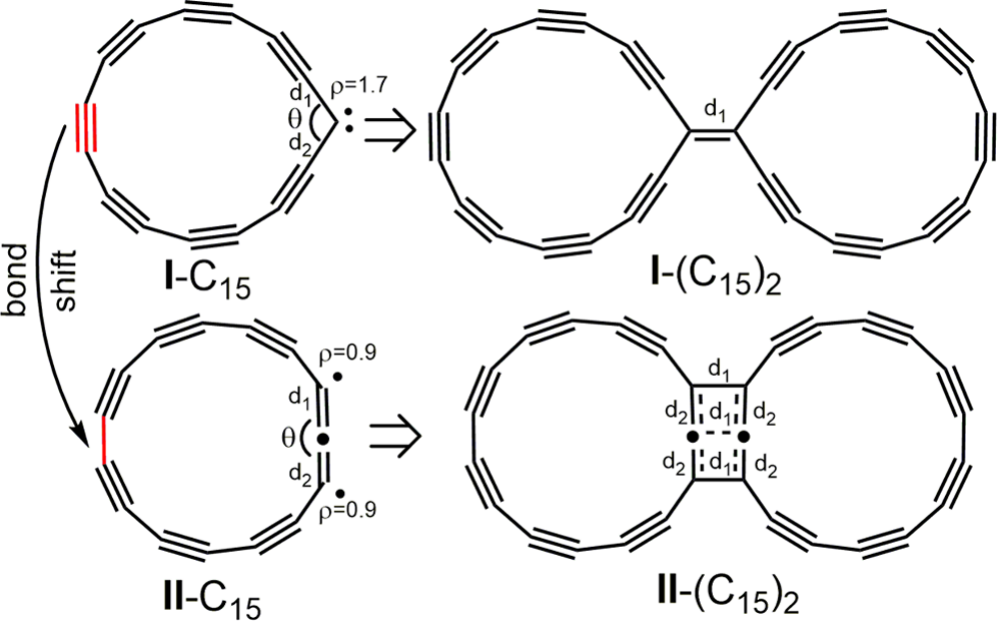
One (I)- and two (II)-center resonance structures of the triplet state of C_15_. The reaction of two C_15_ molecules yielding a connected dimer. The reaction of one (I)- and two (II)-center carbene structures of C_15_ in the triplet state yielding a connected dimer. The same reaction may also occur for two C_19_ molecules in the triplet state.

Despite the fact that the triplet state of the odd-number cyclo[*n*]carbons is generally nonaromatic with an odd number of electrons in the π_in_ and π_out_ subsystems, it has a bond-shift rearrangement between the resonant one (I)- and two (II)-center carbene structures, as seen in Figure 3. A similar bond-shift transformation was previously predicted for the even-number cyclo[*n*]carbons^10,15^ through the cumulenic transition state (TS). For the odd-number cyclo[*n*]carbons C_15_ and C_19_, the TS structure is more complicated, involving a simultaneous change of the θ angle and single–triple bond shifts.

We have successfully optimized both type-I and type-II structures for C_15_ and C_19_ cyclocarbons in the triplet state. Type-I structures are characterized by a considerably small θ angle (near 120°, Table 2) and a spin density predominantly localized on the carbene atom (Figure 3), while type-II structures sustain a θ angle close to 180° (Table 2) and the spin density equally localized on two atoms adjacent to formal carbene centrum (Figure 3).

**Table 2:** Relative Energies (*E*_rel_ in kcal mol^–1^), Magnetically Induced Ring-Current Strengths (*I* in nA T^–1^), and some Selected Structural Parameters (*d*_1_, *d*_2_ in Å and θ in deg) for Type-I and Type-II Resonance Structures of the Triplet State of C_15_ and C_19_ and Their Dimers

molecule	*E*_rel_	*d*_1_	*d*_2_	θ	*I*
I-C_15_^a^	+3.8	1.402	1.402	119.4	1.4^c^
II-C_15_^a^	0	1.286	1.290	173.4	0.6^c^
I-C_19_^a^	+6.5	1.397	1.397	122.9	0.6^c^
II-C_19_^a^	0	1.284	1.286	176.1	0.1^c^
I-(C_15_)_2_^b^	0	1.358			5.8
II-(C_15_)_2_^b^	+37.5	1.478	1.392		1.8
I-(C_19_)_2_^b^	0	1.361			3.9
II-(C_19_)_2_^b^	+38.6	1.477	1.391		1.0

a Calculations at the CASSCF(14;12)/6-31G(d,p) level.

b Calculations at the BHandHLYP/def2-TZVP level.

c Calculated at the BHandHLYP/def2-TZVP level using the CASSCF-optimized molecular structures.

The type-I and type-II resonance structures should thus have different reaction pathways and products. The dimerization of the one-center C_15_ carbene yields a dimer linked via a double bond, whereas dimerization of the two-center structure C_15_ results in a dimer where the two rings are coupled by three CC bonds. It is analogous to the dual behavior of *bis*(9-anthryl)carbene that acts as a two-center biradical when the terminal >C^•^H groups are unprotected, whereas it acts as a one-center carbene when these groups are protected by phenyl substituents (>C^•^Ph).^60^ The type-I dimers of C_15_ and C_19_ are predicted to be globally weakly aromatic, sustaining a magnetically induced ring current flowing through the *d*_1_ bond, whereas the type-II dimers of C_15_ and C_19_ are nonaromatic, sustaining a weak ring current. The rectangular bridge consisting of six carbon atoms sustains local diatropic (“aromatic”) ring currents of 6.7 and 5.9 nA T^–1^ for the dimers of C_15_ and C_1_, respectively (Table S5).

## IV Conclusions

In summary, the electronic and molecular structures of the odd-number cyclo[*n*]carbons with *n* = 5, 7, 9, ..., 29 have been studied at DFT (BHandHLYP) and CASSCF levels of theory. The aromatic character and the degree of aromaticity were analyzed by calculating the magnetically induced current density using the GIMIC method. The calculations show that the DFT level of theory predicts a triplet ground state for large odd-number cyclo[*n*]carbons with *n* > 11. The molecular structure of the triplet state has polyyne-type CC bonds with almost equal bond angles. The CASSCF calculations predict on the other hand a low-lying singlet ground state for the [4k+3] cyclo[*n*]carbons and a quasi-degenerate singlet/triplet ground state for [4k+1] (*k* > 4) rings that have a localized carbene structure in the singlet and triplet states. The singlet state of the [4k+3] cyclo[*n*]carbons is aromatic, whereas the one of [4k+1] cyclo[*n*]carbons is antiaromatic. The ring-current strength decreases for large rings. For large *k* values, the studied cyclo[*n*]carbons are nonaromatic as previously obtained for the even-number cyclo[*n*]carbons.^9,32^ The triplet state of the cyclo[*n*]carbons is practically nonaromatic regardless of the *k* value. The electron delocalization and the magnetically induced ring current avoid the carbene atom in the molecular ring resulting in a through-space aromaticity with an even number of electrons in the in-plane and out-of-plane π subsystems. Depending on the number ([4k] or [4k+2]) of electrons in the out-of-plane π subsystem, the odd-number cyclo[*n*]carbons have alternating aromaticity/antiaromaticity depending on the *k* value. The triplet states of C_15_ and C_19_ may have two nearly degenerate diradical resonance structures leading to different reaction pathways of the dimerization reaction. The transition between the resonance structures involves a single–triple bond shift as also previously obtained for even-number cyclo[*n*]carbons.^10,15^

## Supporting Information Available

Computational details and corresponding references on the employed methods, structural parameters, plots of magnetically induced ring currents, and optimized Cartesian coordinates (PDF)
