# Supplementary material for: Odd-Number Cyclo[n]Carbons Sustaining Alternating Aromaticity
Source: J Phys Chem A. 2022 Apr 14;126(16):2445–52. doi: 10.1021/acs.jpca.1c08507 (PMC9059118; doi:10.1021/acs.jpca.1c08507)
Supplement: Supplementary file 1 — jp1c08507_si_001.pdf [file jp1c08507_si_001.pdf]

Odd-number cyclo[n]carbons sustaining alternating aromaticity

Glib V. Baryshnikov,[a,b,*] Rashid R. Valiev,[c*] Lenara I. Valiulina,[d] Alexandr E. Kurtsevich,[d] Theo Kurtén,[c] Dage Sundholm,[c] Michael Pittelkow,[e] Jinglai Zhang[a] and Hans Ågren[a,f,*]

[a] *College of Chemistry and Chemical Engineering, Henan University, Kaifeng, Henan 475004, P.R. China*

[b] *Linköping University, Department of Science and Technology, Laboratory of Organic Electronics, Norrköping, SE-60174 Sweden*

[c] *Department of Chemistry, Faculty of Science, University of Helsinki, FIN-00014, Helsinki, Finland*

[d] *Department of Optics and Spectroscopy, Tomsk State University, Tomsk, 634050, Russia*

[e] *Department of Chemistry, University of Copenhagen, Copenhagen Ø, DK-2100, Denmark*

[f] *Department of Physics and Astronomy, Uppsala University, Uppsala, SE-75120 Sweden*

E-mail: glib.baryshnikov@liu.se, rashid.valiev@helsinki.fi, hans.agren@physics.uu.se

Supporting Information

Table of content

**Table S1.** The energy difference (ΔE_{ST}) between the lowest singlet and triplet states of C_{17} calculated at DFT and CASSCF levels of theory. Different functionals and active spaces were employed in the DFT and CASSCF calculations, respectively.	**S2**
**Table S2**. The ΔEST energy difference calculated at different DFT levels using the CASSCF optimized geometries for cyclocarbons C5-C29. The 6-31G(d,p) basis sets were used.	**S2**
**Table S3.** The molecular structures of the singlet and triplet states of cyclocarbons C5-C29 calculated at two levels of theory. The total energy values are also given for each structure.	**S3**
**Table S4.** The magnetically induced current density of the singlet and triplet states of C_5-C_{29} calculated at the BHandHLYP/def2-TZVP level with the GIMIC method using the molecular structures optimized at the CASSCF(14,12)/6-31G(d,p) level.	**S13**
**Table S5.** The magnetically induced current density and the ring-current strengths for the type I and type II dimers of C_{15} and C_{19} calculated at the BHLYP/def2-TZVP level with the GIMIC method.	**S20**
**Table S6.** Cartesian coordinates for optimized molecular structures of the singlet and triplet states of C_5-C_{29} optimized at the BHandHLYP and CASSCF levels of theory.	**S21**

Table S1. The energy difference (ΔE_{ST}) between the lowest singlet and triplet states of C_{17} calculated at DFT and CASSCF levels of theory. Different functionals and active spaces were employed in the DFT and CASSCF calculations, respectively.

	DFT					CASSCF			
	TPSS (0%HF)	B3LYP (20%HF)	BMK (40%HF)	BHLYP (50%HF)	M06HF (100%HF)	(4,4)	(6,6)	(10,10)	(14,12)
C_{17}	+4.3	+4.1	+6.5	+15.1	+14.3	-12.6	-7.4	-9.9	-1.4

Table S2. The ΔE_{ST} energy difference (in kcal mole^{-1}) calculated at different DFT levels using the CASSCF optimized geometries for cyclocarbons C_5-C_{29}. The 6-31G(d,p) basis set was used.

	CASSCF	BHandHLYP	B3LYP	O3LYP	M062X	wB97XD
C_5	-16	-5.5	-7.7	-6.1	-9.1	-8.3
C_7	-20.3	50.3	4.5	4.0	-0.1	3.9
C_9	-8.8	32.4	7.0	9.4	7.3	8.0
C_{11}	-40.2	24.1	-29.7	-21.1	-27.2	-32.4
C_{13}	-5.6	54.1	-5.1	-4.5	-1.9	-2.5
C_{15}	-27.3	39.1	-17.3	-18.5	-11.3	-11.7
C_{17}	-1.4	55.8	-0.28	0.1	1.4	2.3
C_{19}	-25.4	38.3	-13.6	-15.3	-7.5	-7.3
C_{21}	-0.1	-29.3	2.2	2.4	3.6	5.1
C_{23}	-24.2	-0.58	-10.7	-12.6	-5.2	-4.4
C_{25}	0.8	11.1	3.7	3.7	5.1	6.8
C_{27}	-15.2	-0.86	-11.3	-13.5	-5.5	-4.5
C_{29}	-1.9	11.6	2.2	1.3	5.1	7.1

Table S3. The molecular structures of the singlet and triplet states of cyclocarbons C_5-C_{29} calculated at two levels of theory. The total energy values are also given for each structure.

CASSCF(14,12)/6-31G(d,p)	BHandHLYP/def2-TZVP
C_5 Singlet	
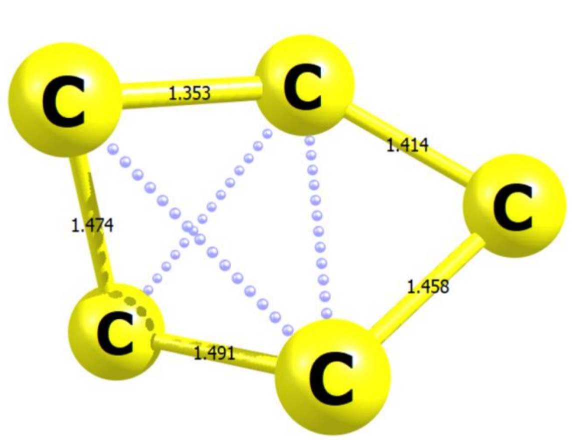	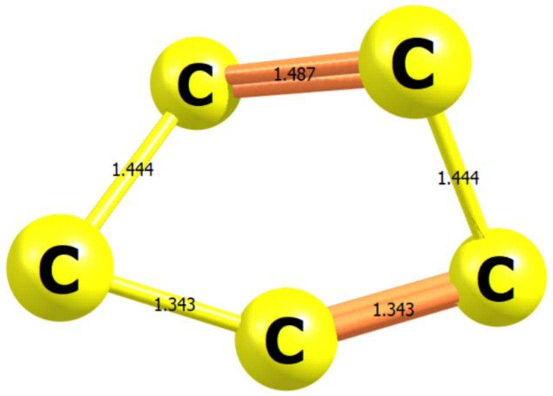
-189.108283 a.u.	-190.0228
C_5 Triplet	
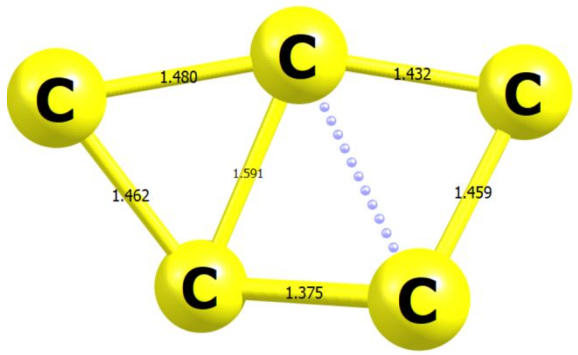	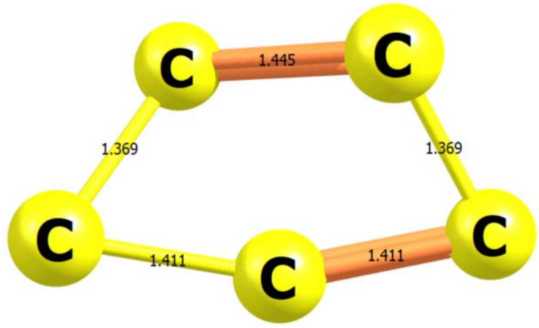
-189.083	-190.020
C_7 Singlet	
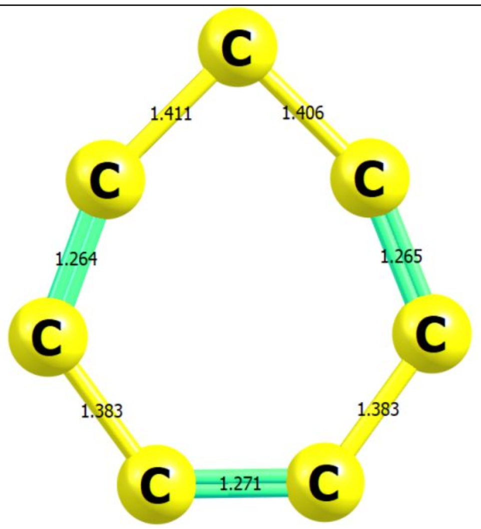	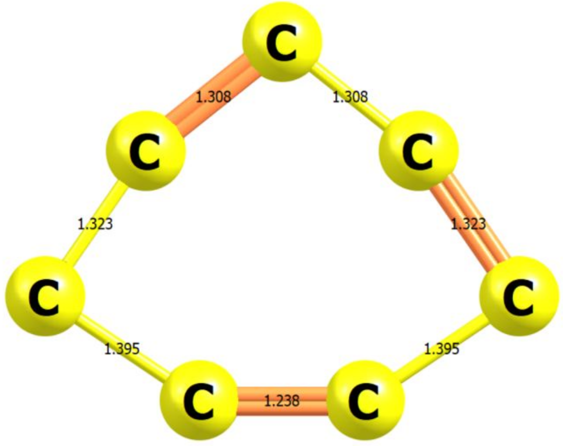
-264.8534	-266.2378
C_7 Triplet	

<table>
<tr><td>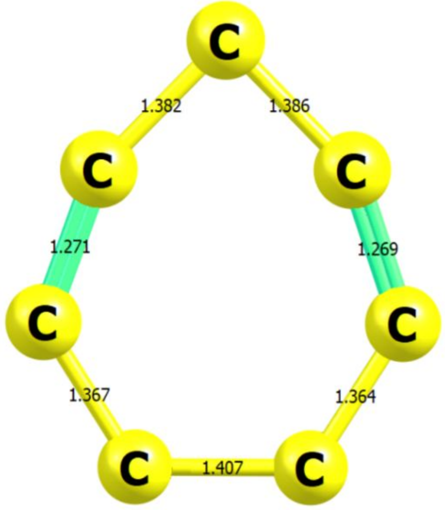
</td><td>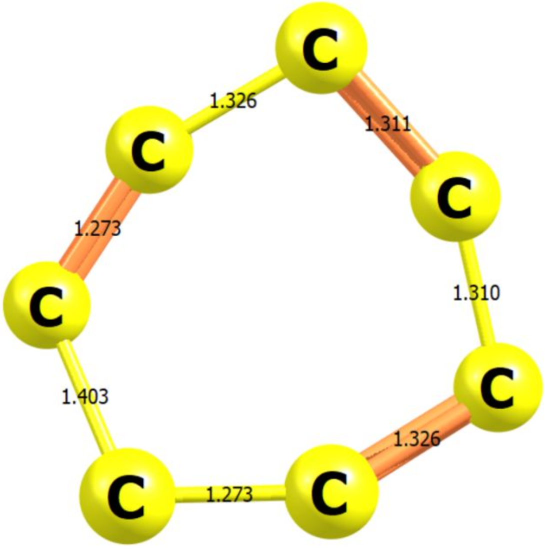
</td></tr>
<tr><td>-264.821</td><td>-266.211</td></tr>
<tr><td colspan="2">C_9 Singlet</td></tr>
<tr><td>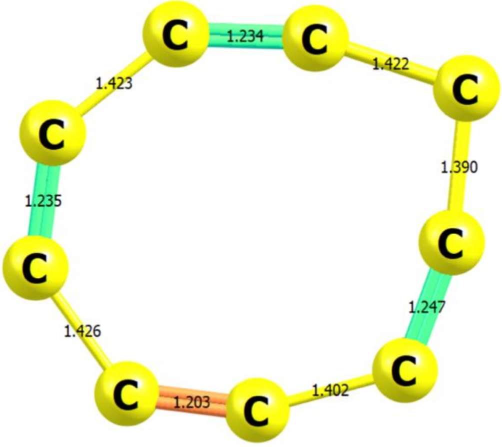
</td><td>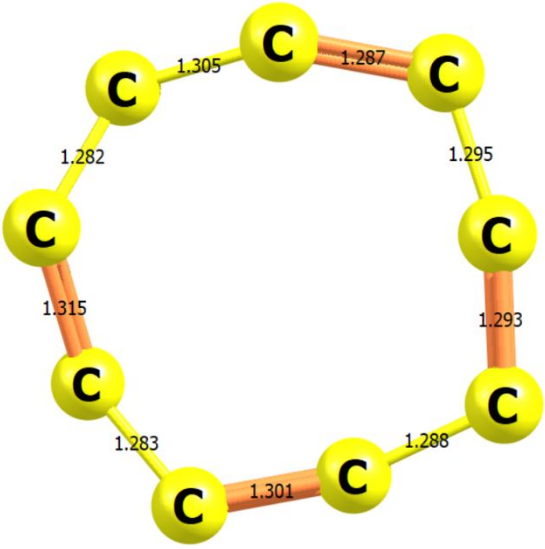
</td></tr>
<tr><td>-340.52494</td><td>-342.3811</td></tr>
<tr><td colspan="2">C_9 Triplet</td></tr>
<tr><td>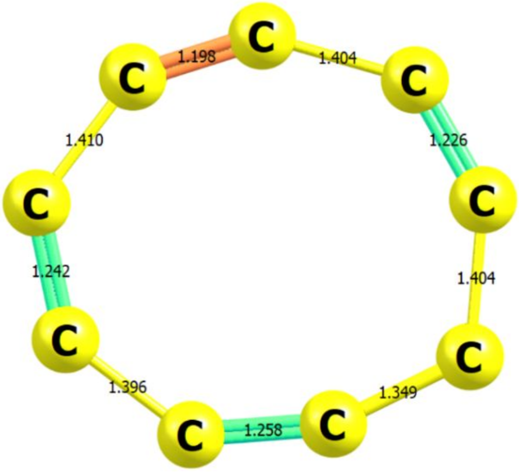
</td><td>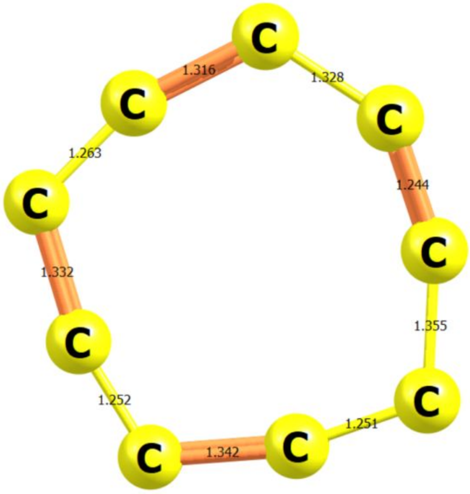
</td></tr>
<tr><td>-340.511</td><td>-342.379</td></tr>
</table>

<table>
<tr><td colspan="2">C_{11} Singlet</td></tr>
<tr><td>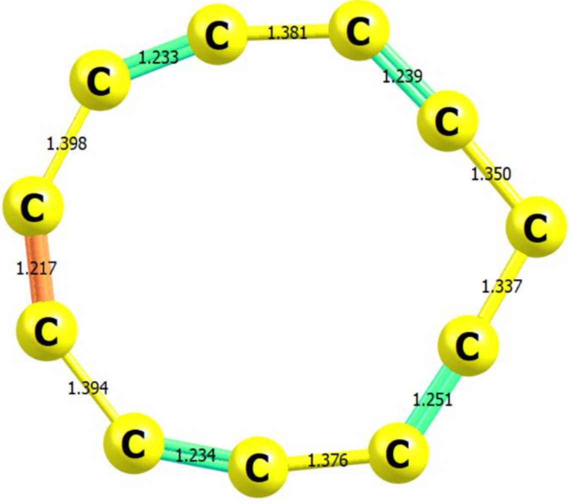
</td><td>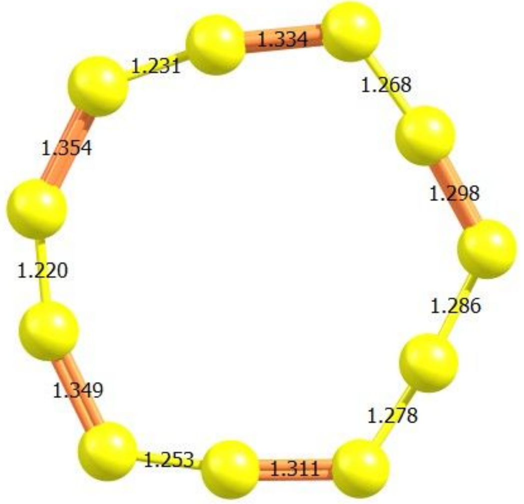
</td></tr>
<tr><td>-416.232508</td><td>-418.5707</td></tr>
<tr><td colspan="2">C_{11} Triplet</td></tr>
<tr><td>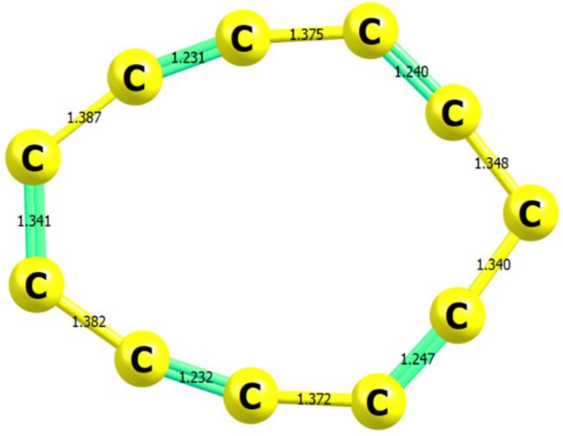
</td><td>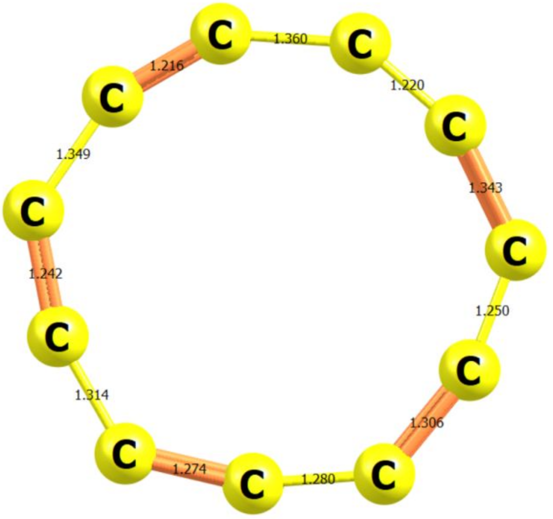
</td></tr>
<tr><td>-416.169</td><td>-418.562</td></tr>
<tr><td colspan="2">C_{13} Singlet</td></tr>
<tr><td>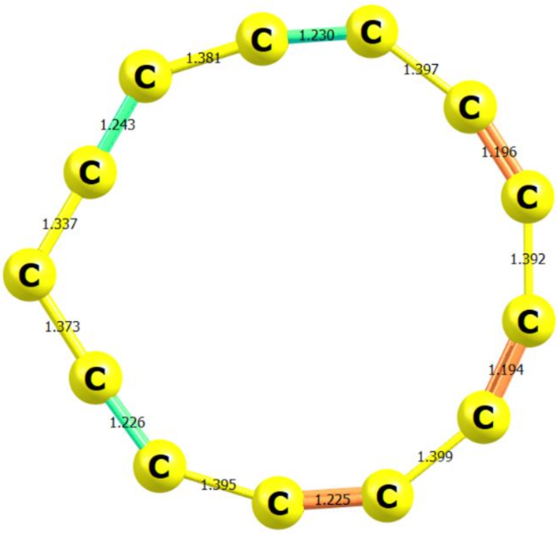
</td><td>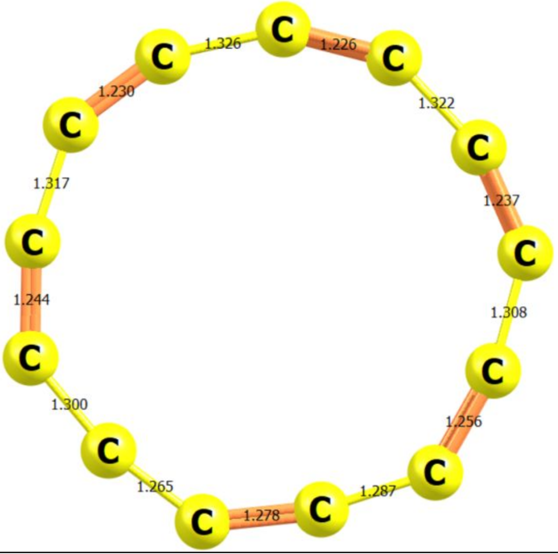
</td></tr>
<tr><td>-491.887652</td><td>-494.7081</td></tr>
</table>

C_{13} Triplet	
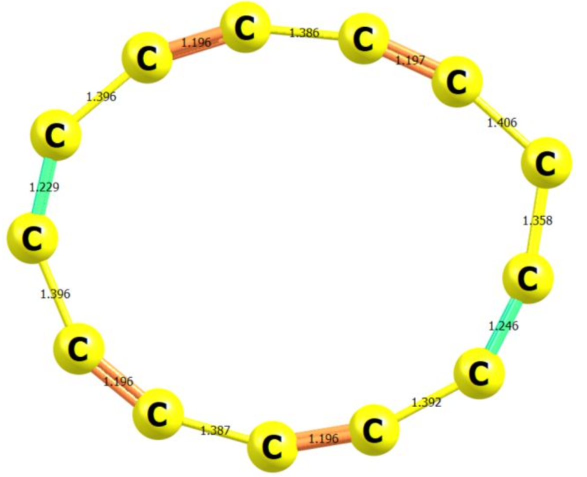	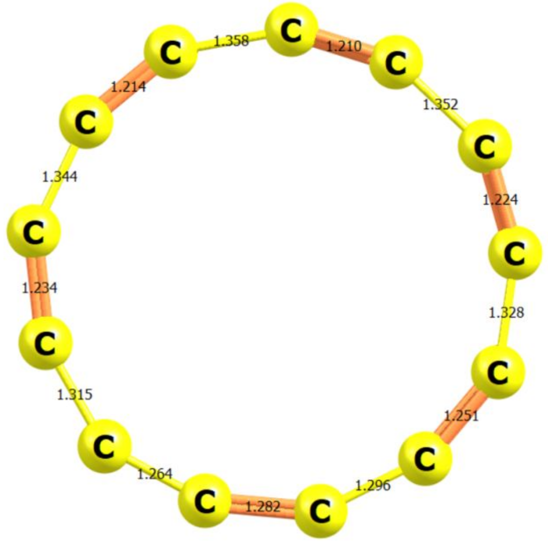
-491.879	-494.722
C_{15} Singlet	
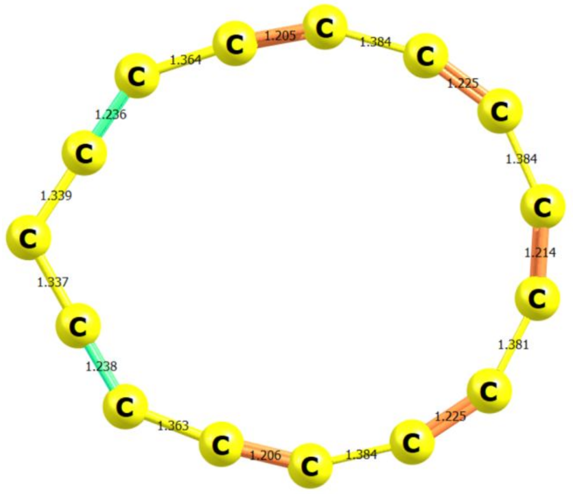	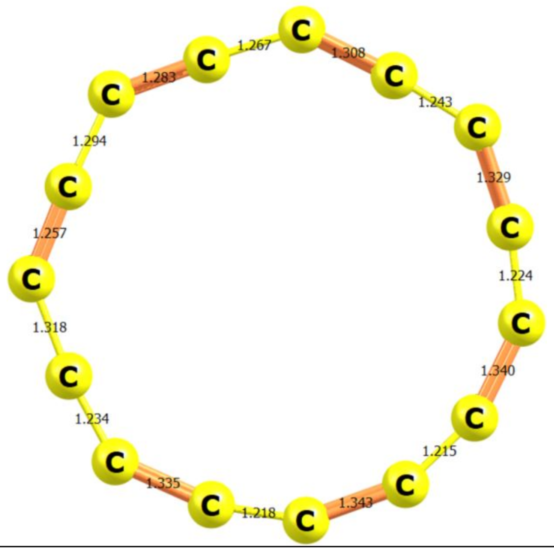
-567.602189	-570.8742
II-C_{15} Triplet	
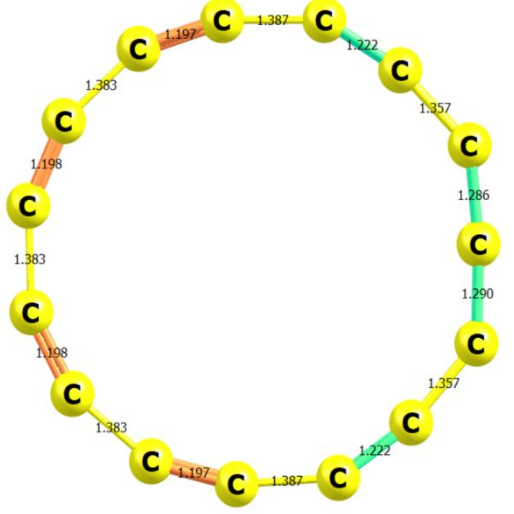	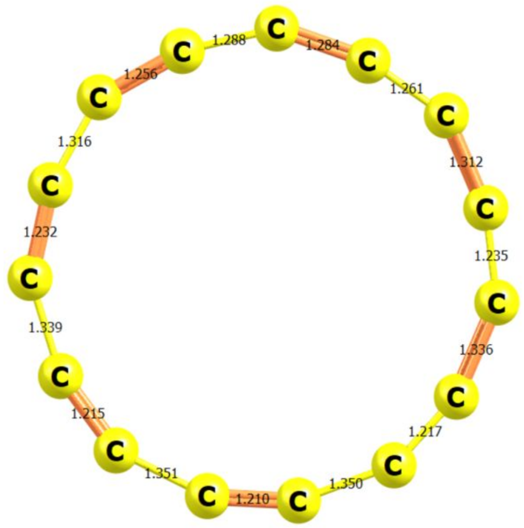
-567.565	-570.883

<table>
<tr><td>I-C_{15} Triplet</td><td>-</td></tr>
<tr><td>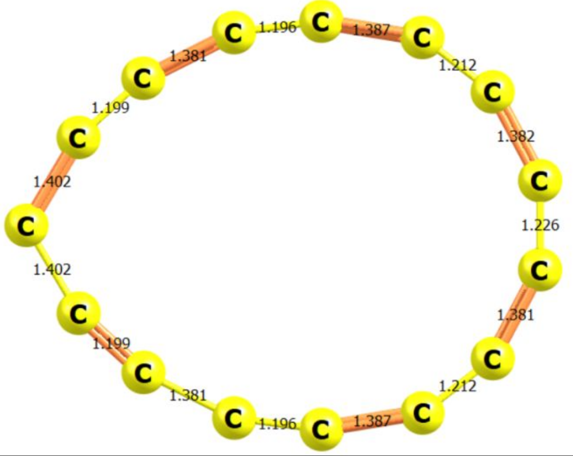
</td><td>-</td></tr>
<tr><td>-567.559</td><td></td></tr>
<tr><td colspan="2">C_{17} Singlet</td></tr>
<tr><td>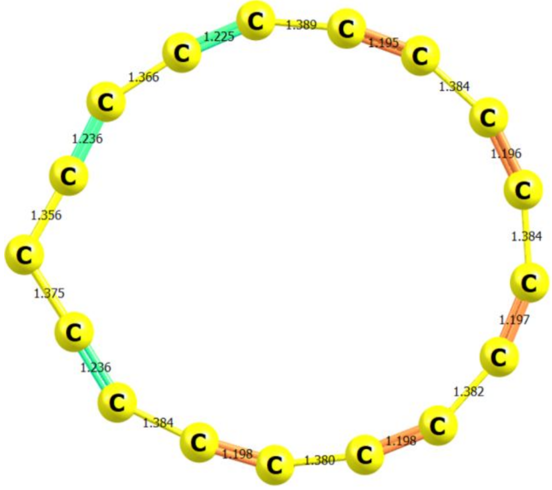
</td><td>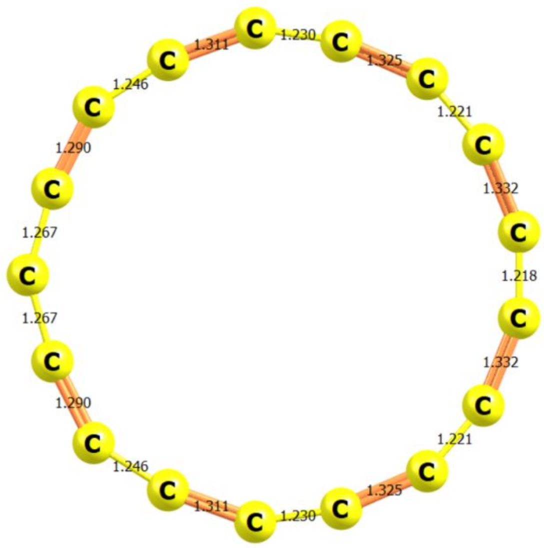
</td></tr>
<tr><td>-643.267993</td><td>-647.0115</td></tr>
<tr><td colspan="2">C_{17} Triplet</td></tr>
<tr><td>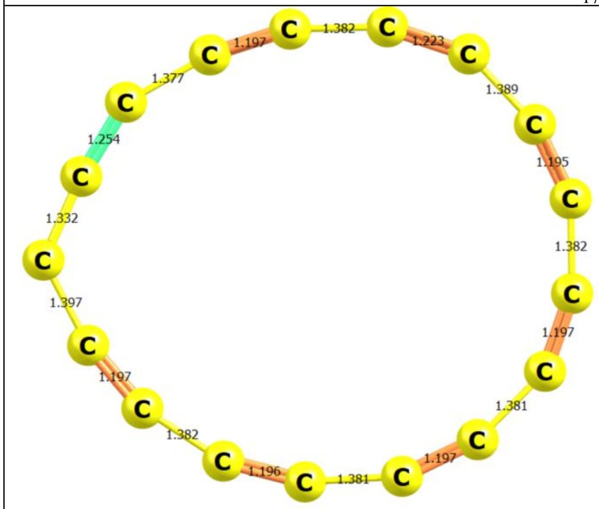
</td><td>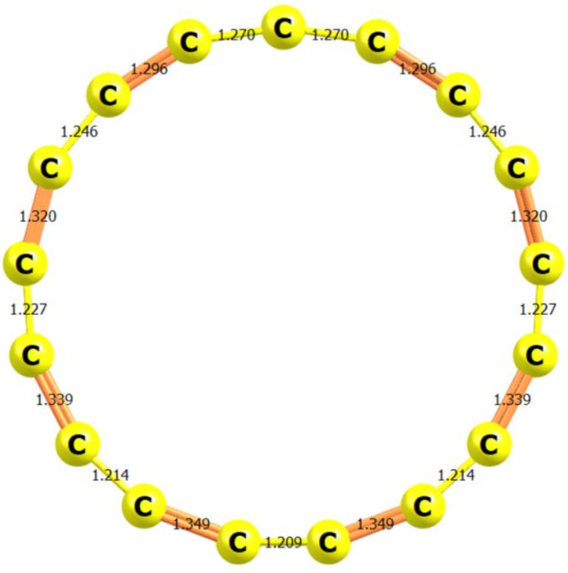
</td></tr>
<tr><td>-643.2657248</td><td>-647.036</td></tr>
</table>

<table>
<tr><td colspan="2">C_{19} Singlet</td></tr>
<tr><td>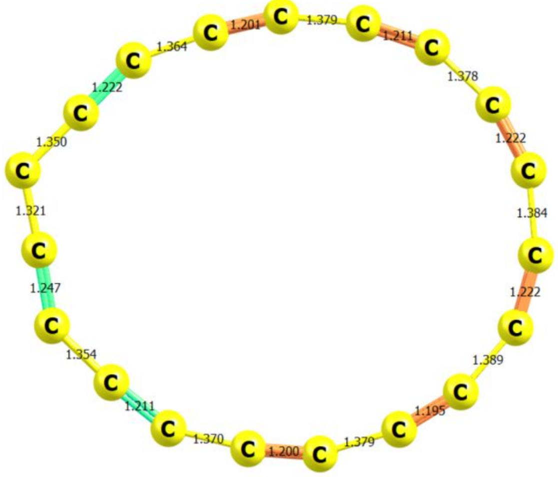
</td><td>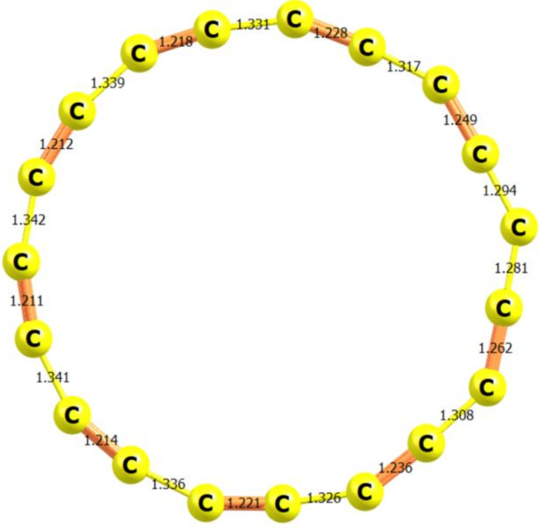
</td></tr>
<tr><td>-718.978302</td><td>-723.1665</td></tr>
<tr><td colspan="2">II-C_{19} Triplet</td></tr>
<tr><td>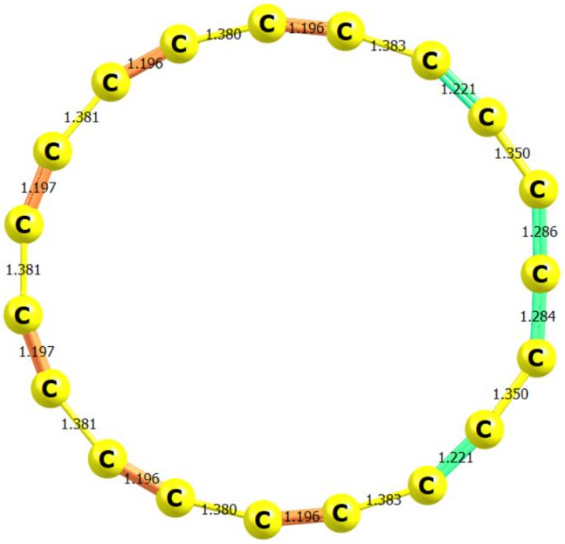
</td><td>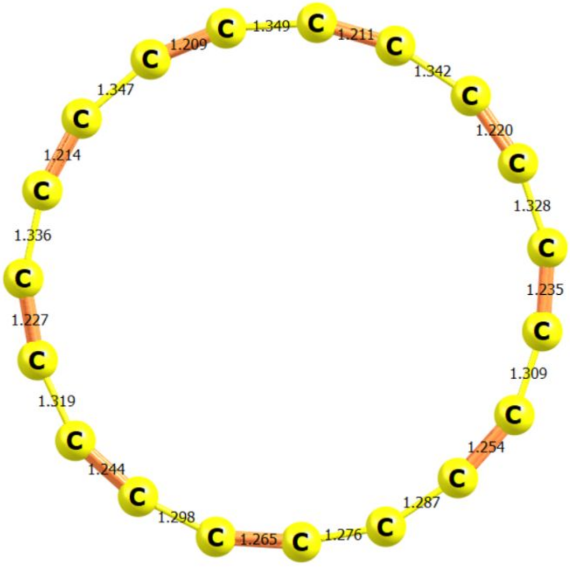
</td></tr>
<tr><td>-718.948</td><td>-723.186</td></tr>
<tr><td>I-C_{19} Triplet</td><td></td></tr>
<tr><td>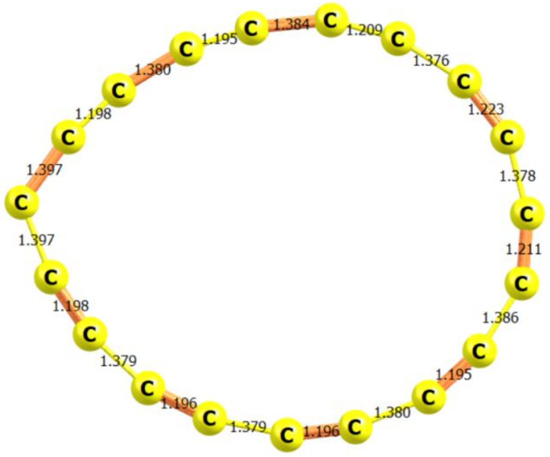
</td><td></td></tr>
<tr><td>-718.938</td><td></td></tr>
<tr><td colspan="2">C_{21} Singlet</td></tr>
</table>

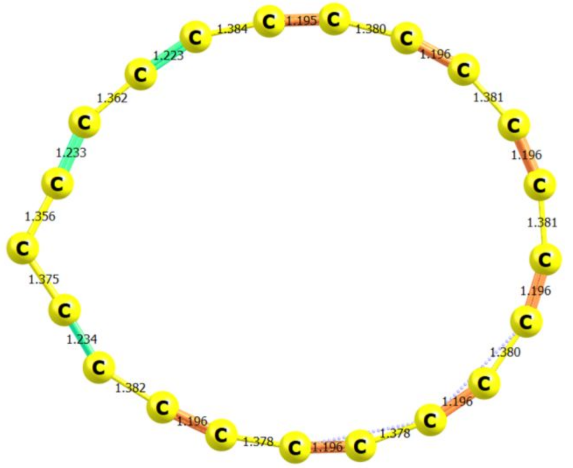	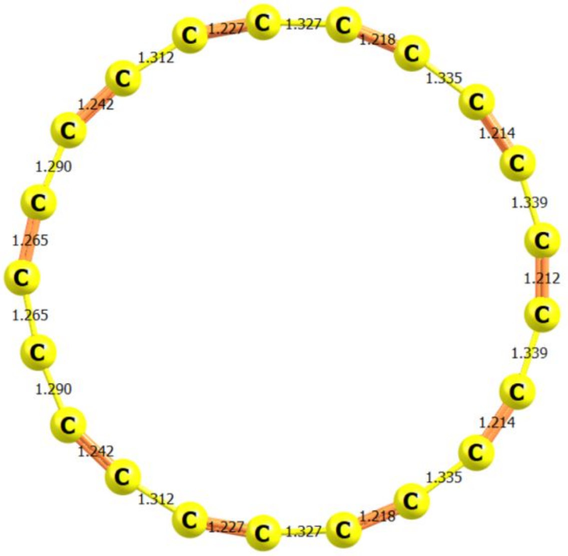
-794.645223	-799.3042
C_{21} Triplet	
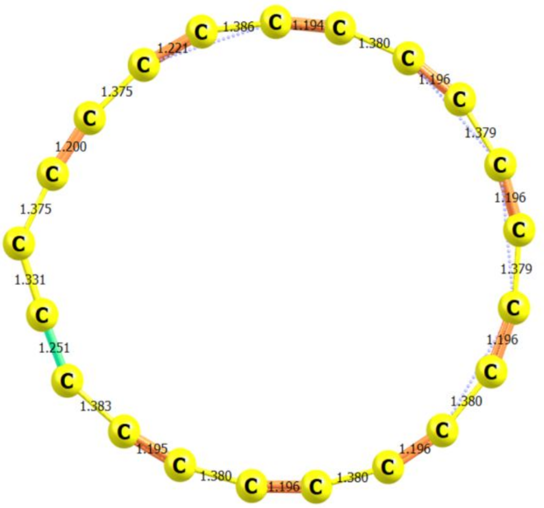	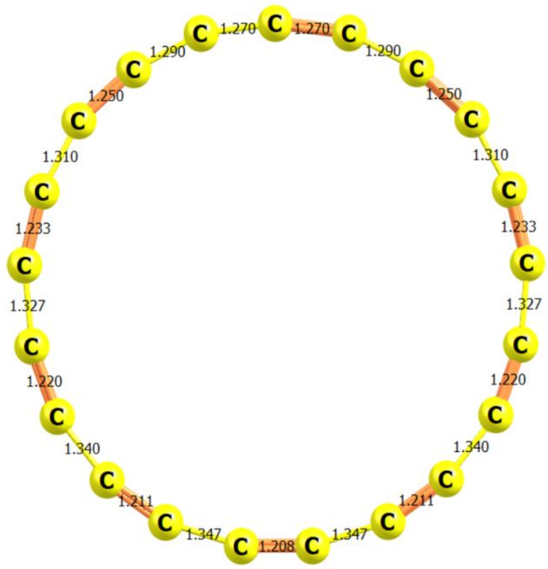
-794.645	-799.335
C_{23} Singlet	
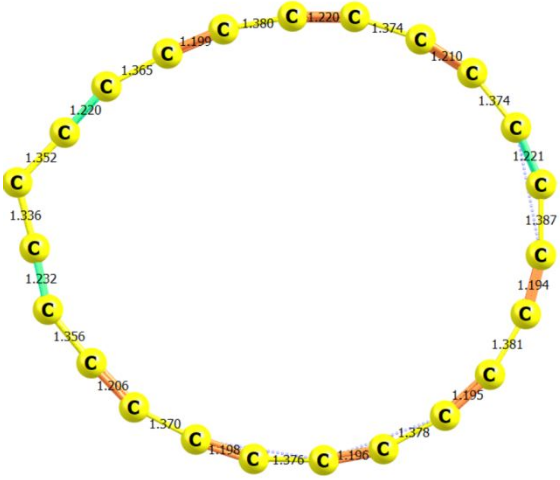	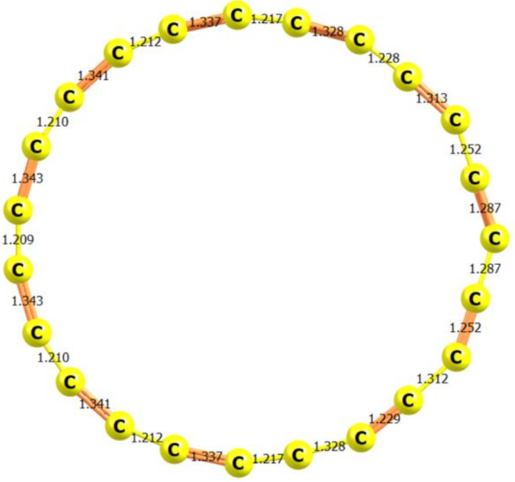
-870.349325	-875.4539

<table>
<tr><td colspan="2">C_{23} Triplet</td></tr>
<tr><td>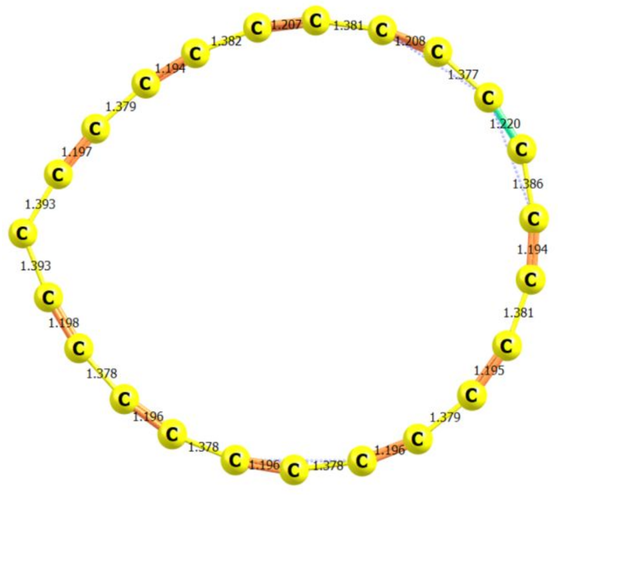
</td><td>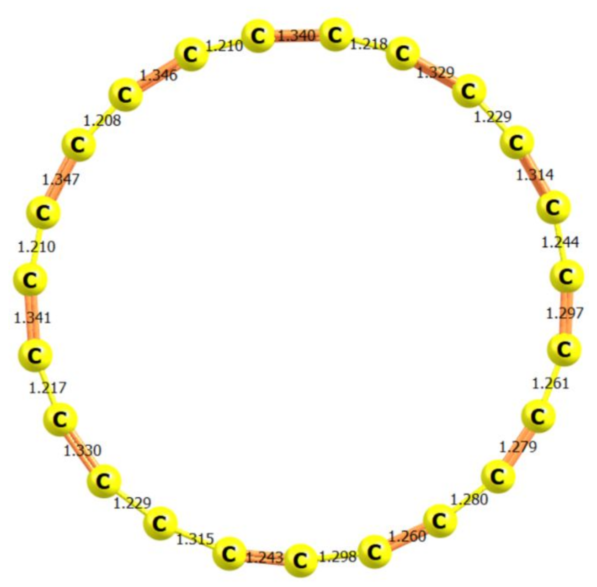
</td></tr>
<tr><td>-870.311</td><td>-875.481</td></tr>
<tr><td colspan="2">C_{25} Singlet</td></tr>
<tr><td>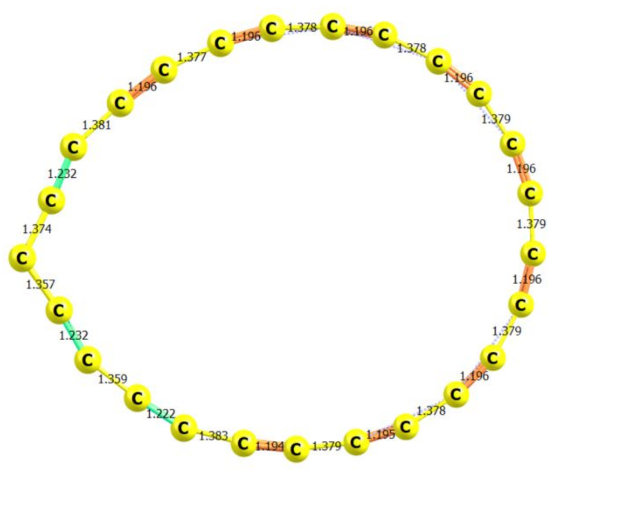
</td><td>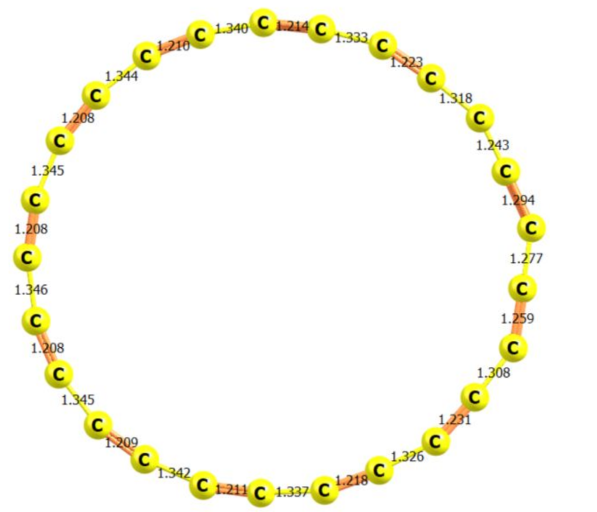
</td></tr>
<tr><td>-946.017404</td><td>-951.5953</td></tr>
<tr><td colspan="2">C_{25} Triplet</td></tr>
<tr><td>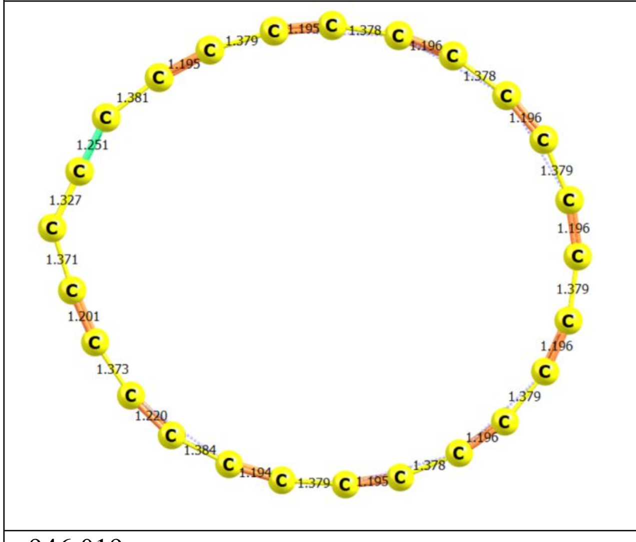
</td><td>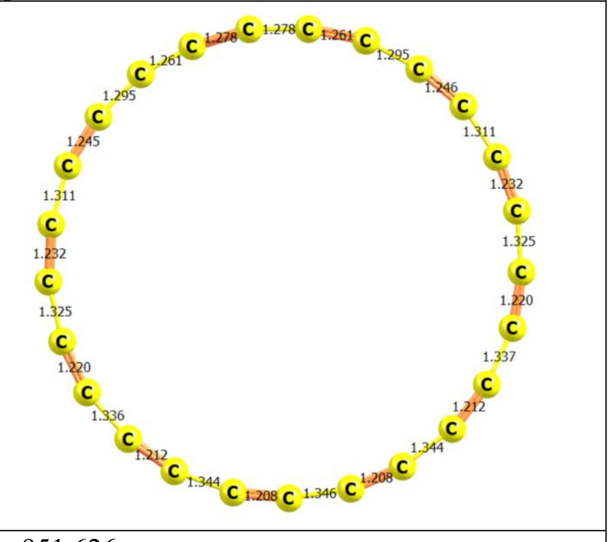
</td></tr>
<tr><td>-946.019</td><td>-951.626</td></tr>
</table>

C_{27} Singlet	
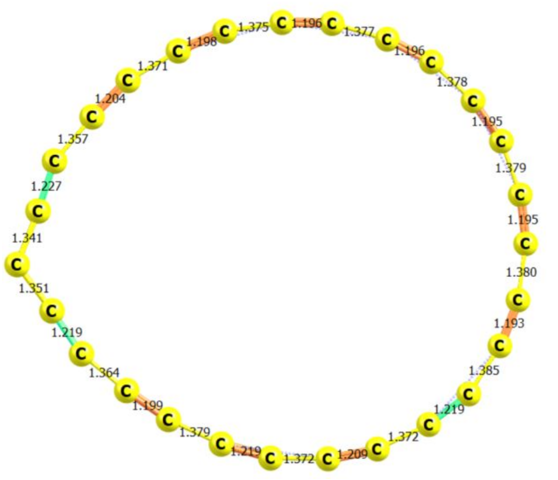	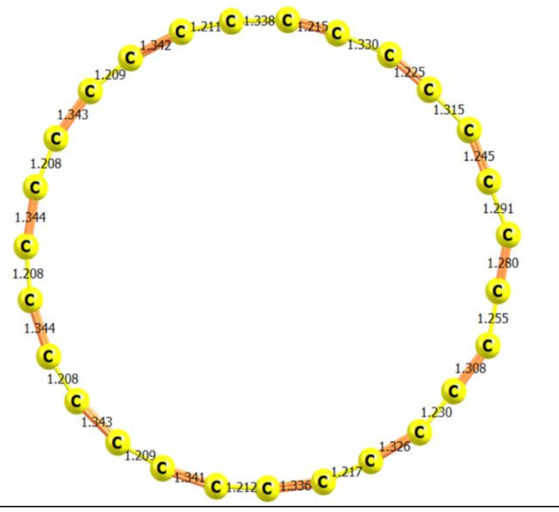
-1021.71788	-1027.7384
C_{27} Triplet	
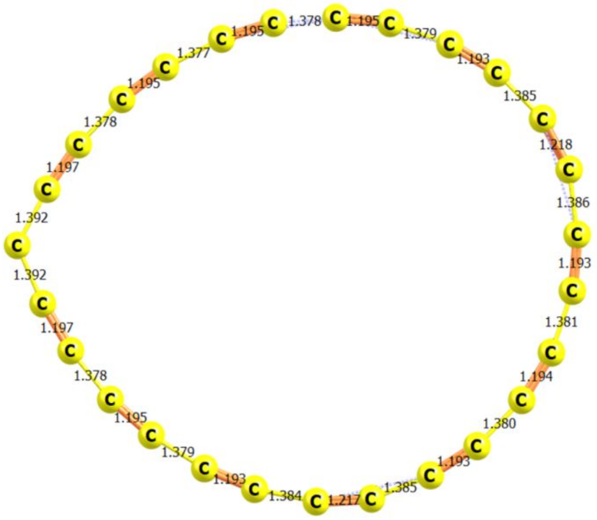	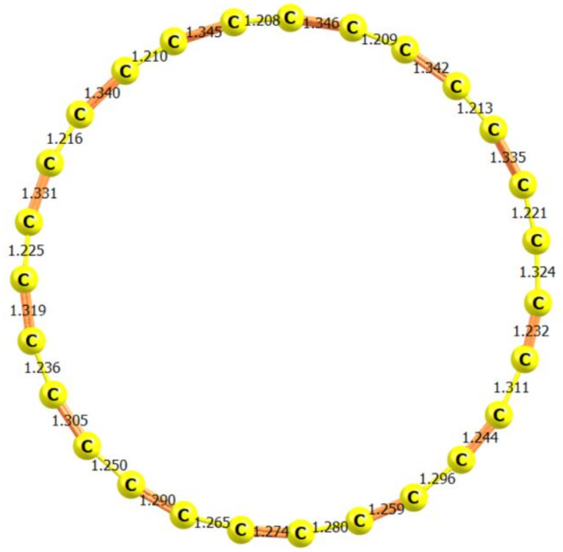
-1021.690	-1027.770
C_{29} Singlet	
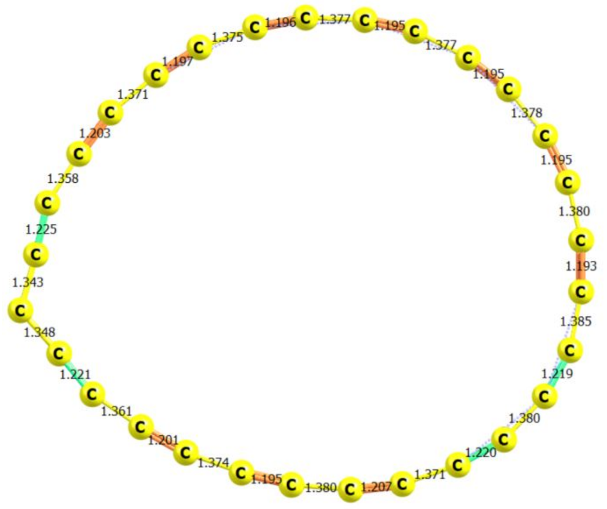	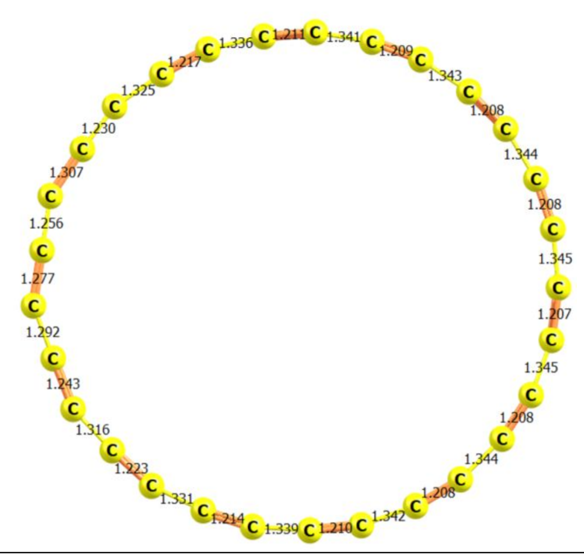
-1097.403	-1103.8794

C$_{29}$ Triplet	
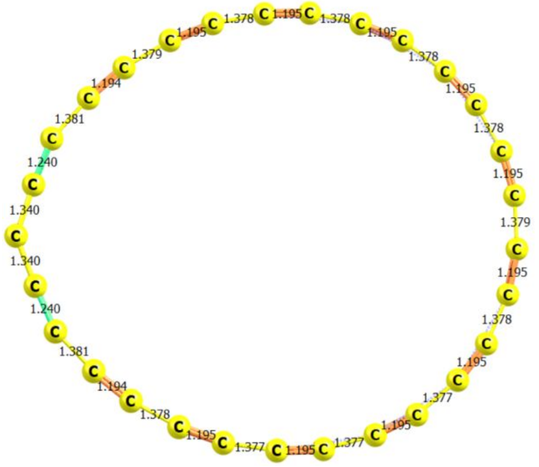	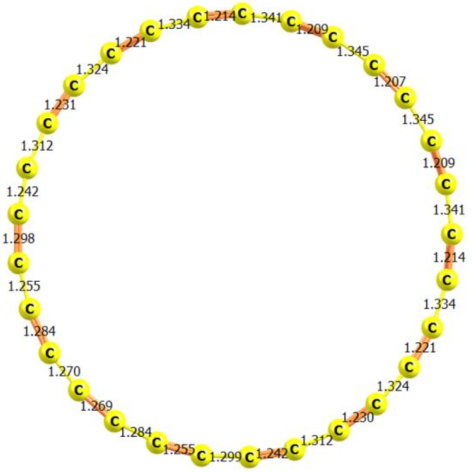
-1097.400	-1103.913

Table S4. The magnetically induced current density of the singlet and triplet states of C_5-C_{29} calculated at the BHandHLYP/def2-TZVP level with the GIMIC method using the molecular structures optimized at the CASSCF(14,12)/6-31G(d,p) level.

Top view	Side view
C_5 Singlet	
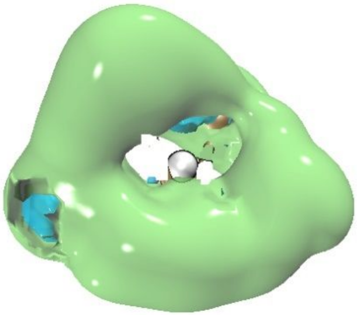	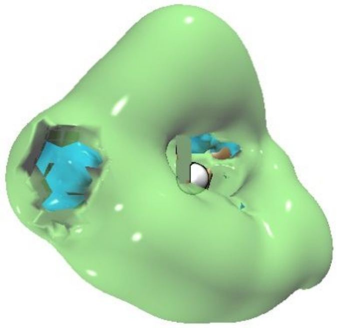
C_5 Triplet	
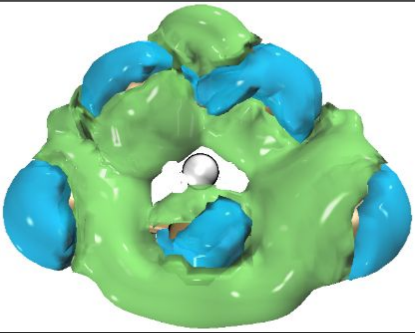	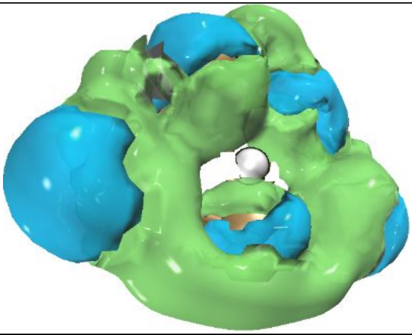
C_7 Singlet	
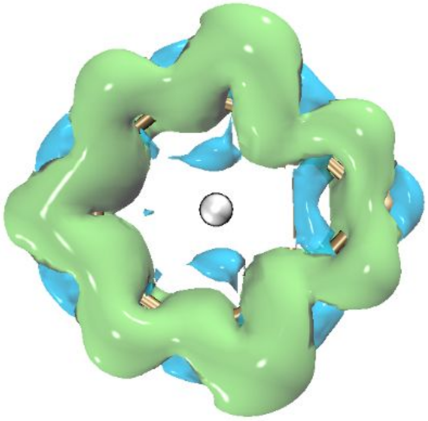	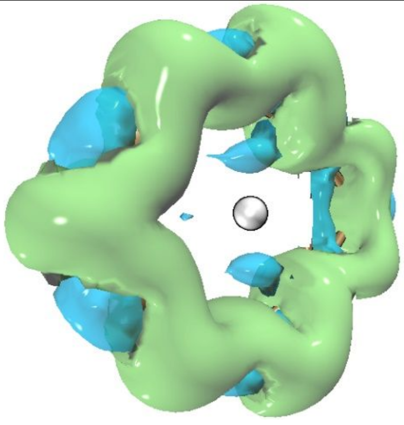
C_7 Triplet	
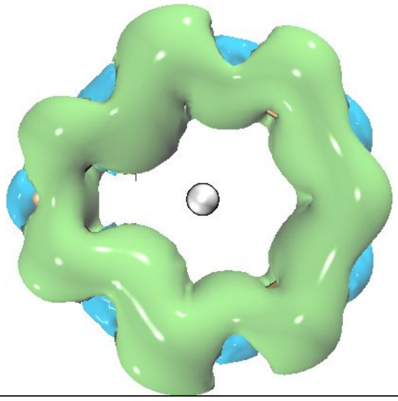	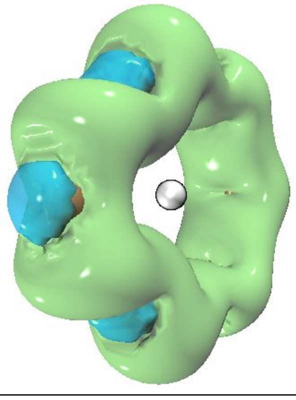
C_9 Singlet	

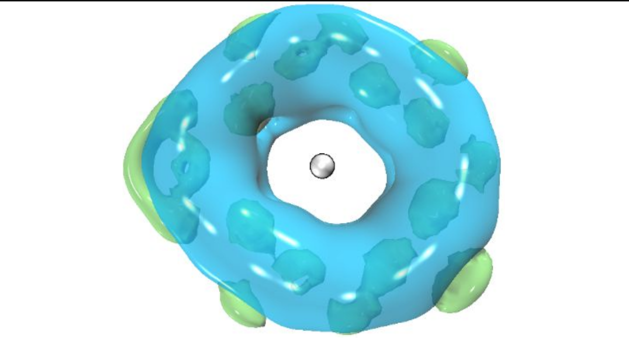
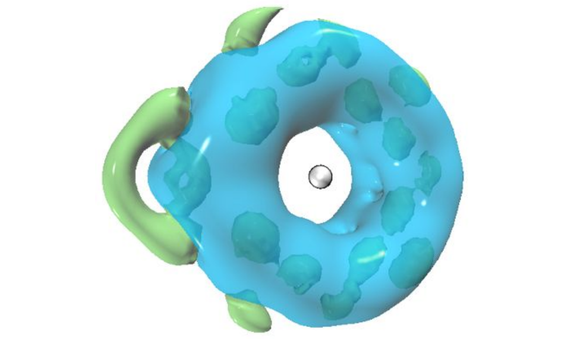

C_9 Triplet

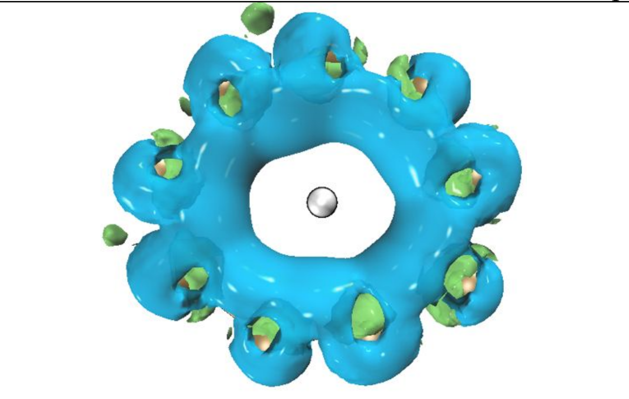
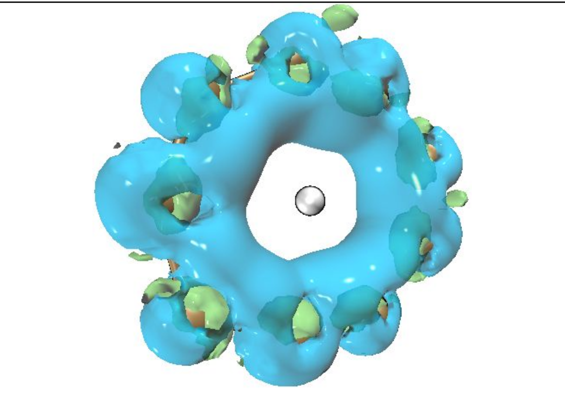

C_{11} Singlet

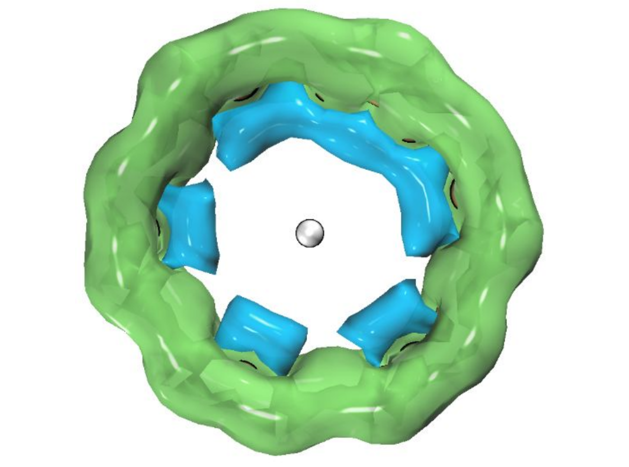
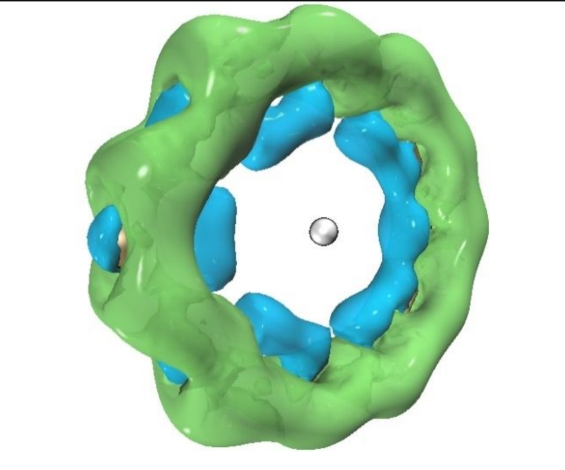

C_{11} Triplet

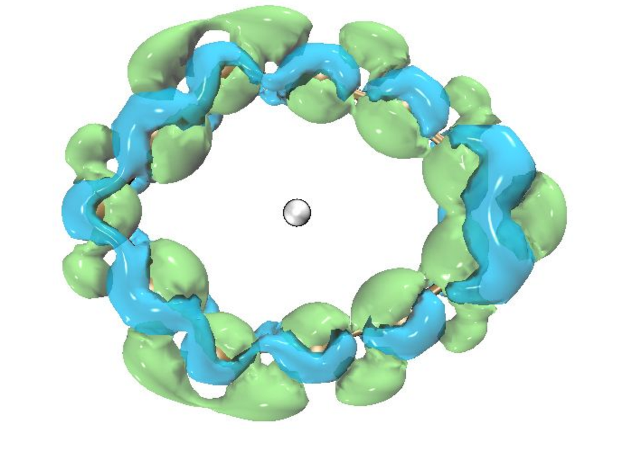
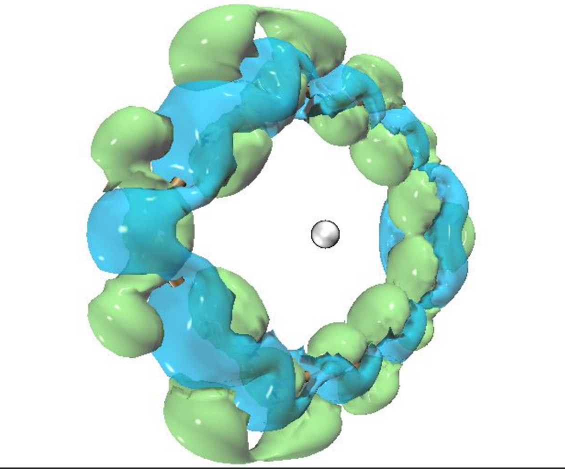

C_{13} Singlet

<table>
<tr><td>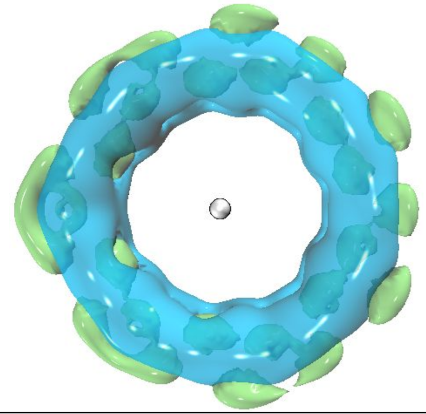</td><td>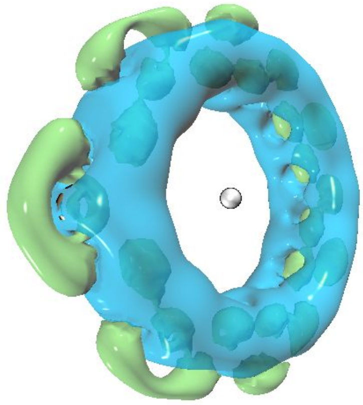</td></tr>
<tr><td colspan="2">C_{13} Triplet</td></tr>
<tr><td>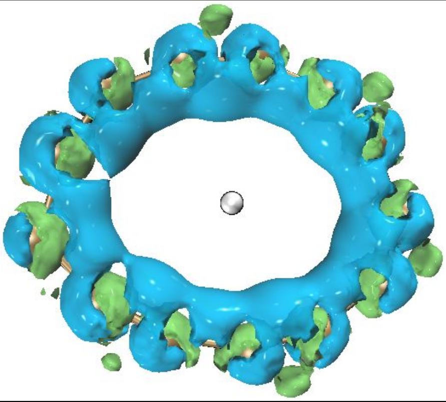</td><td>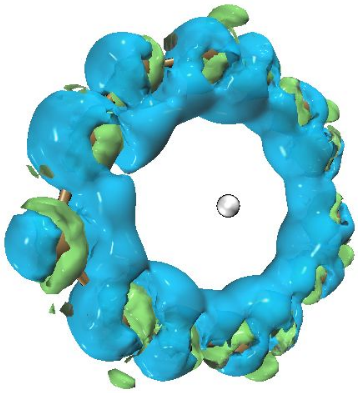</td></tr>
<tr><td colspan="2">C_{15} Singlet</td></tr>
<tr><td>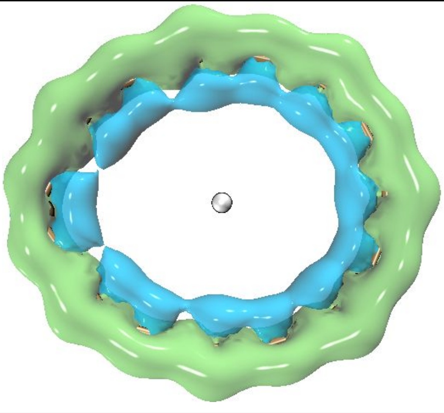</td><td>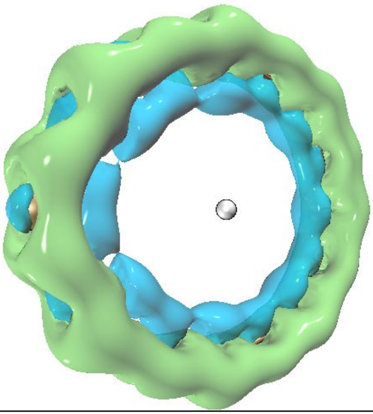</td></tr>
<tr><td colspan="2">I-C_{15} Triplet</td></tr>
<tr><td>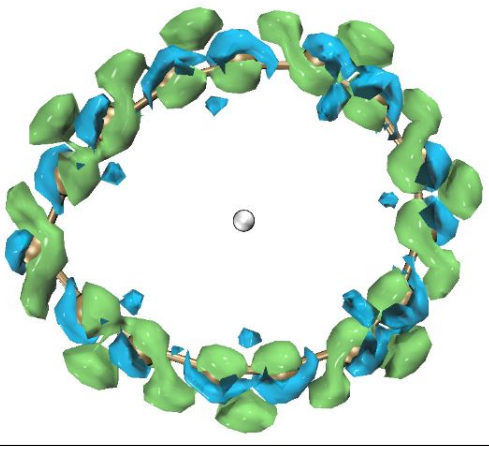</td><td>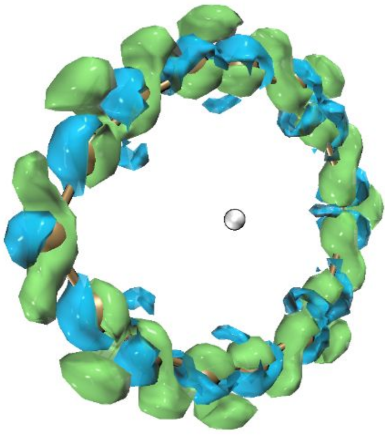</td></tr>
<tr><td colspan="2">II-C_{15} Triplet</td></tr>
</table>

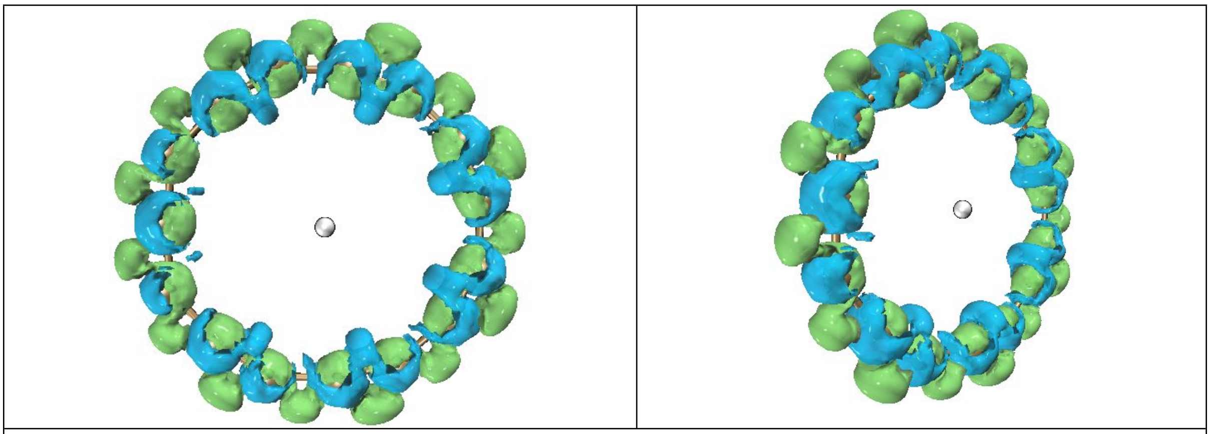

C_{17} Singlet

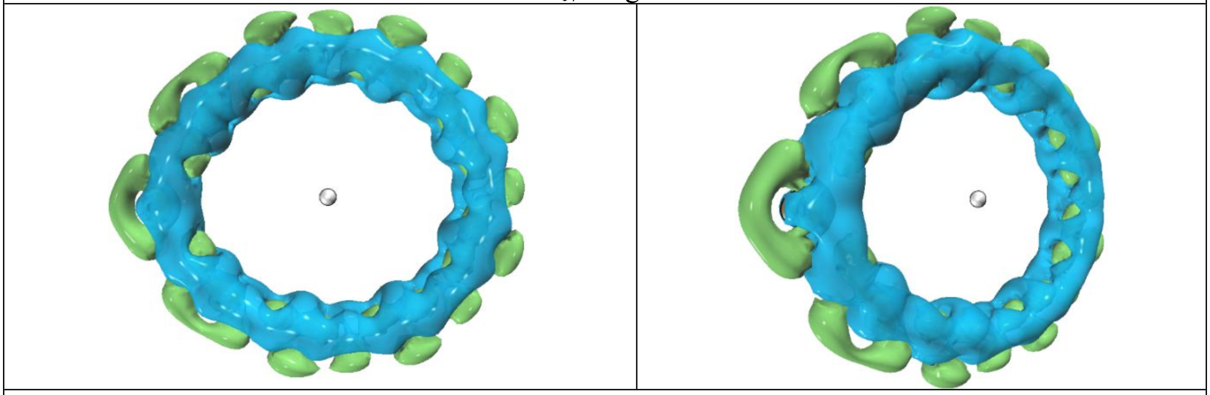

C_{17} Triplet

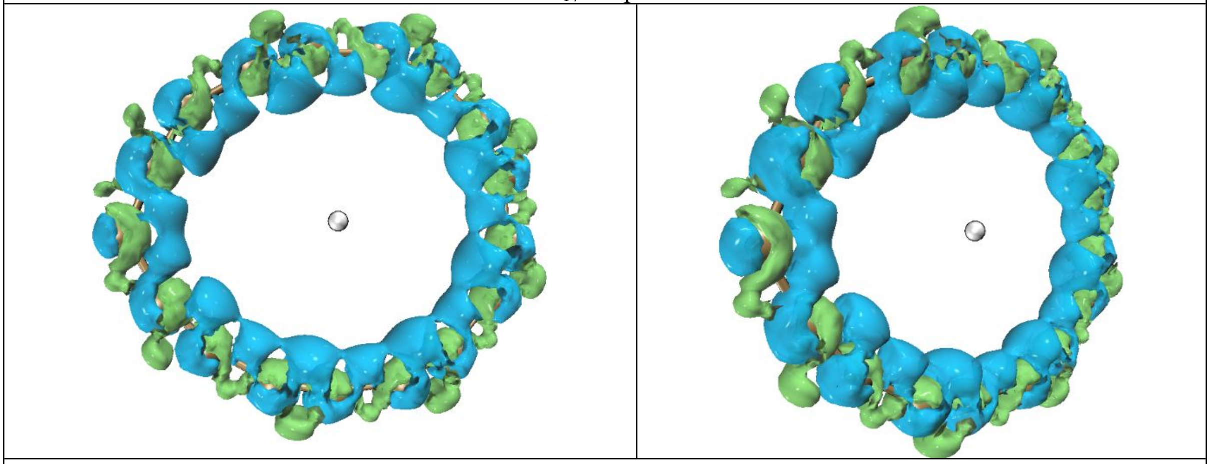

C_{19} Singlet

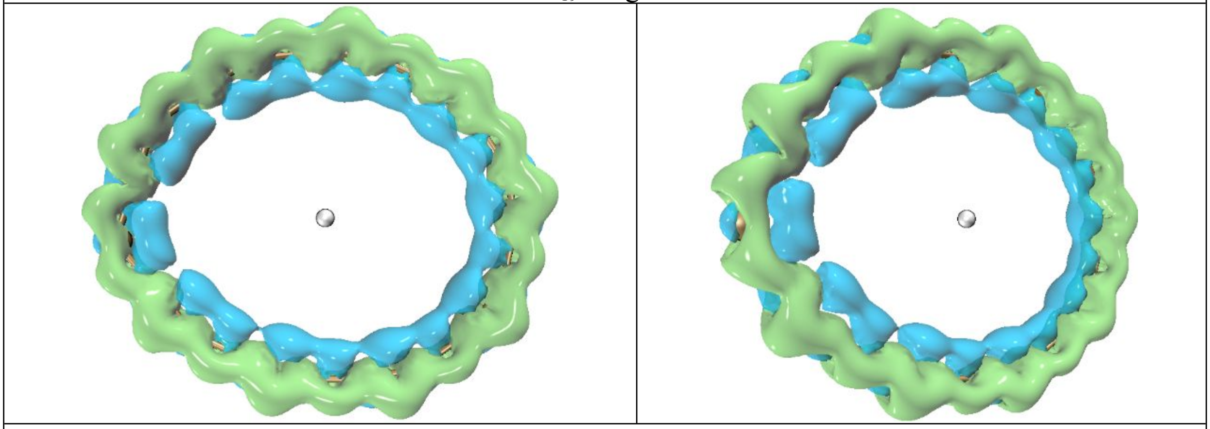

I-C_{19} Triplet

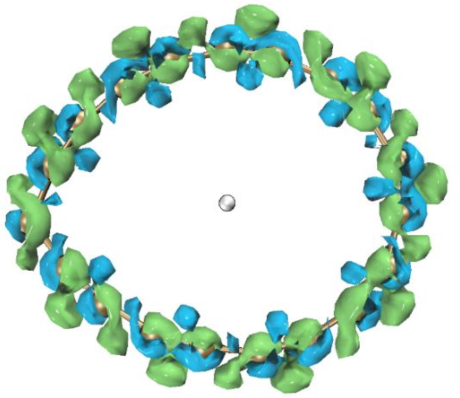
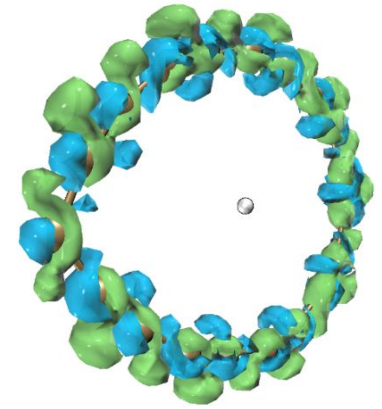

II-C_{19} Triplet

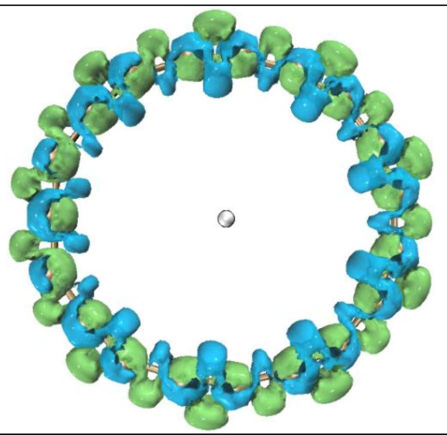
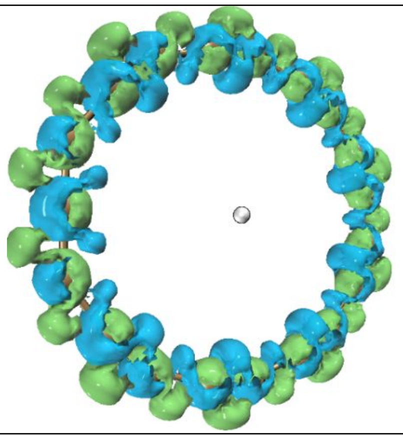

C_{21} Singlet

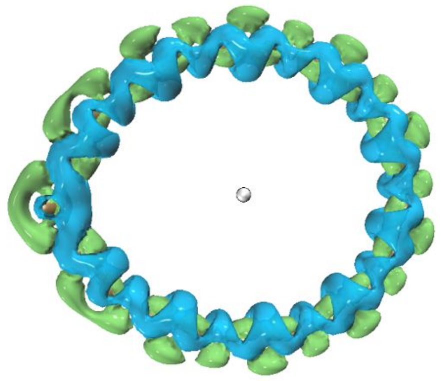
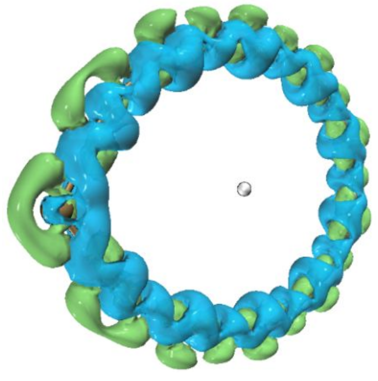

C_{21} Triplet

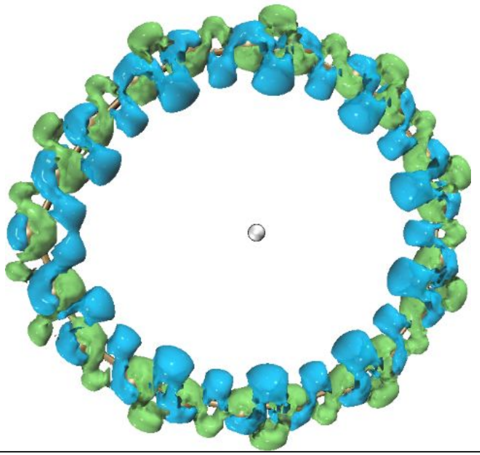
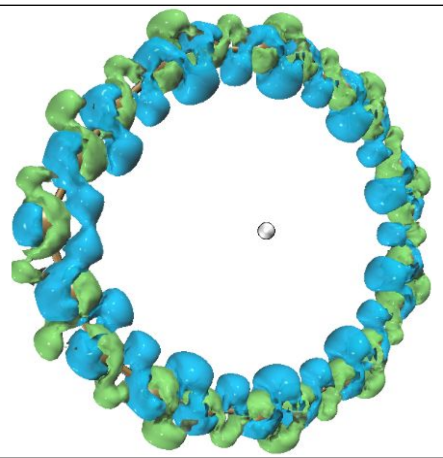

C_{23} Singlet

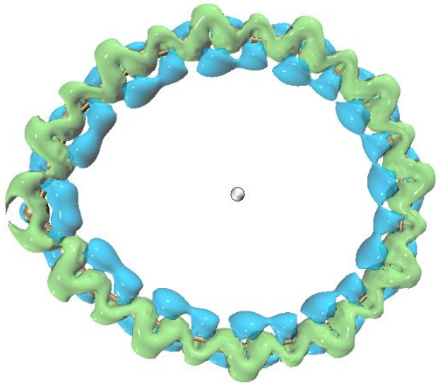

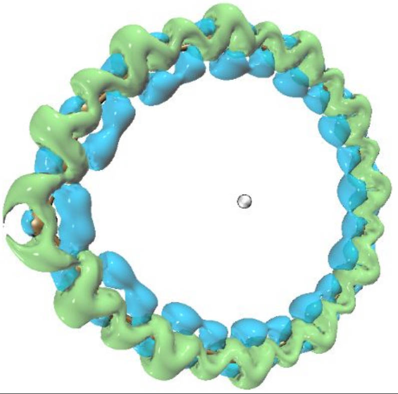

C_{23} Triplet

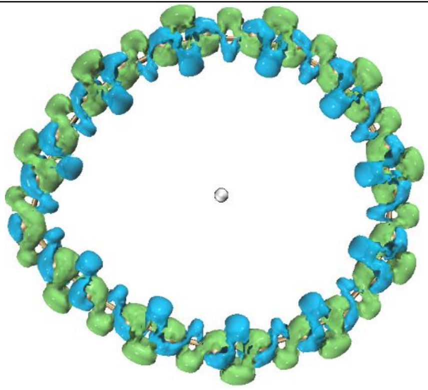

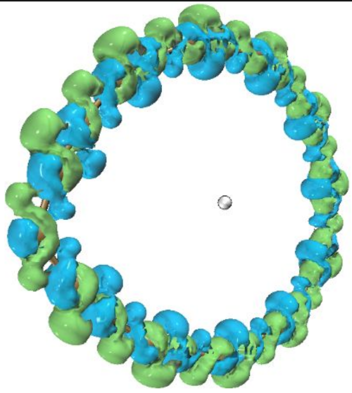

C_{25} Singlet

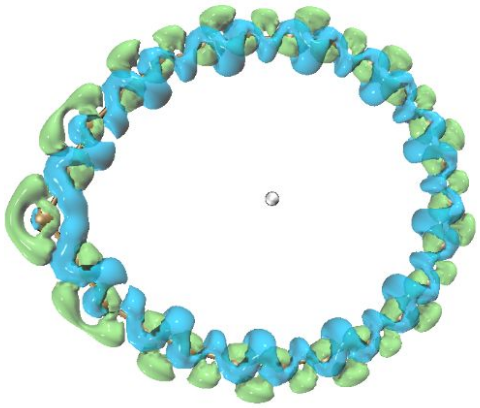

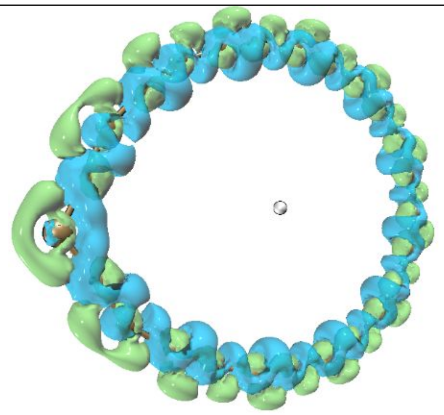

C_{25} Triplet

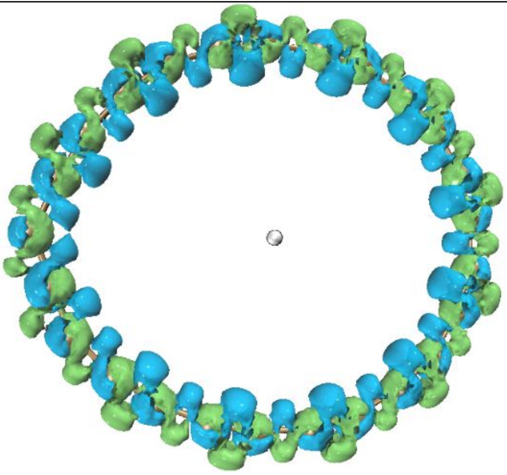

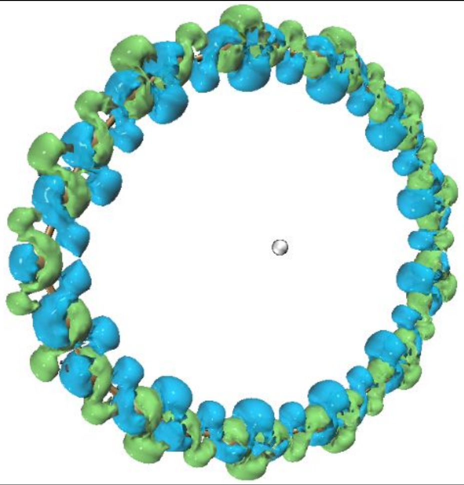

C_{27} Singlet

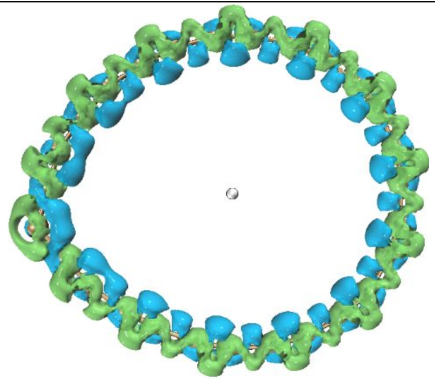
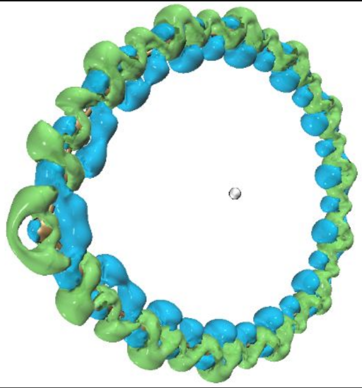

C_{27} Triplet

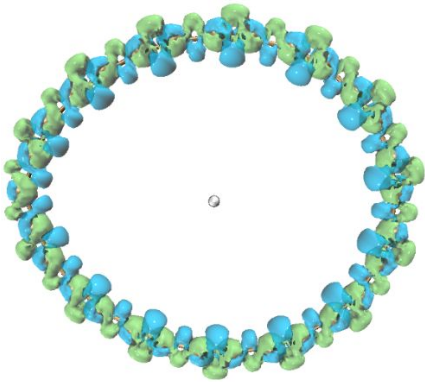
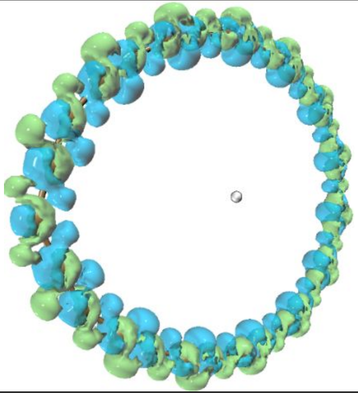

C_{29} Singlet

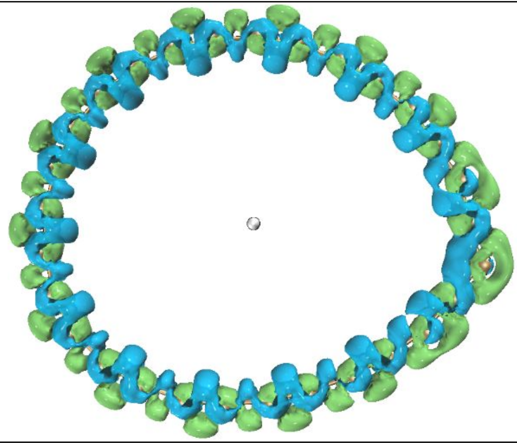
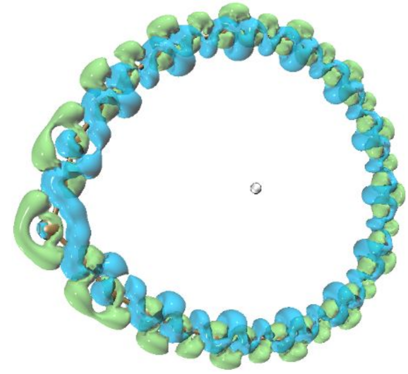

C_{29} Triplet

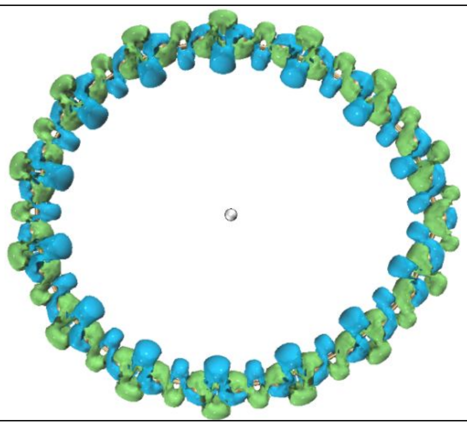
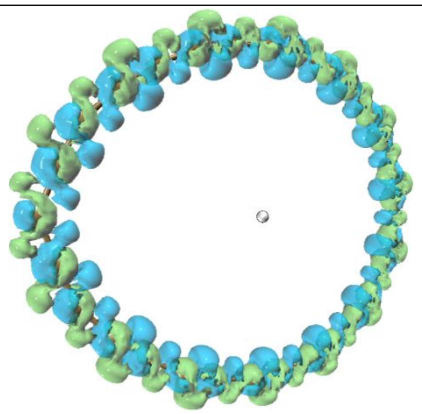

Table S5. The magnetically induced current density and the ring-current strengths for the type I and type II dimers of C_{15} and C_{19} calculated at the BHLYP/def2-TZVP level with the GIMIC method.

<table>
<tr><td colspan="2">Dimer of C_{15} single</td></tr>
<tr><td>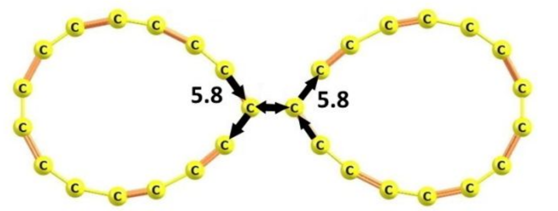
</td><td>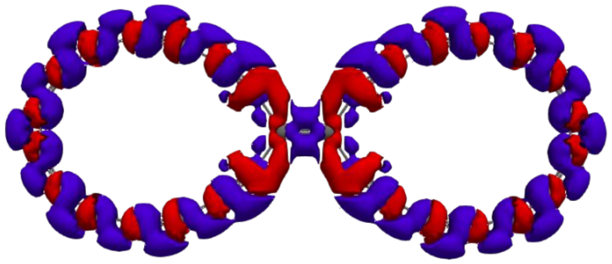</td></tr>
<tr><td colspan="2">Dimer of C_{15} triplet</td></tr>
<tr><td>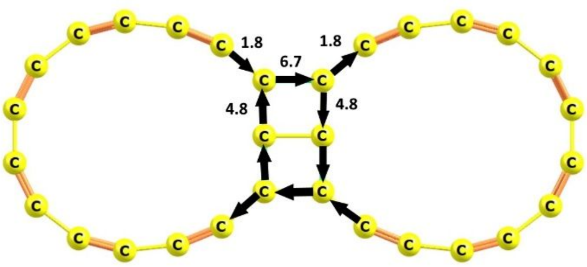
</td><td>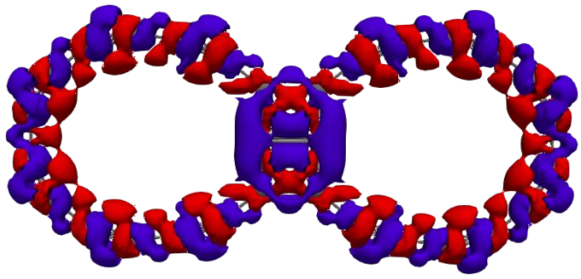</td></tr>
<tr><td colspan="2">Dimer of C_{19} singlet</td></tr>
<tr><td>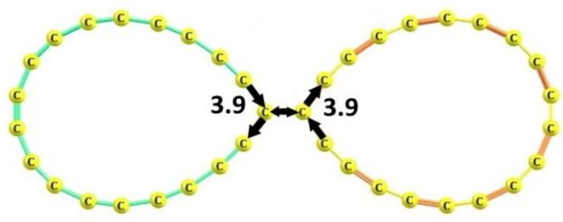
</td><td>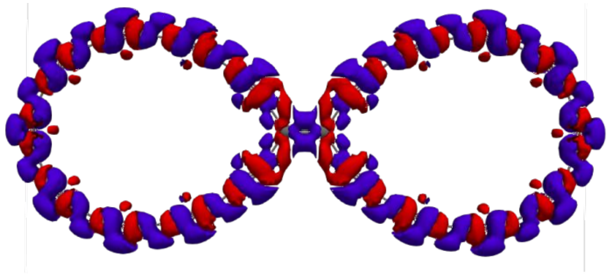</td></tr>
<tr><td colspan="2">Dimer of C_{19} triplet</td></tr>
<tr><td>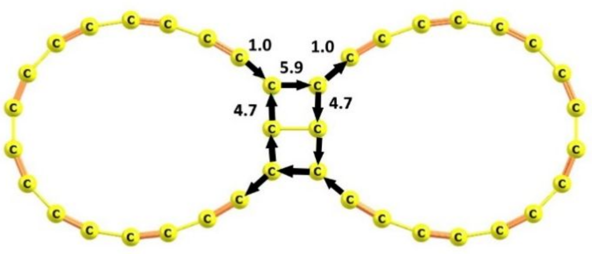
</td><td>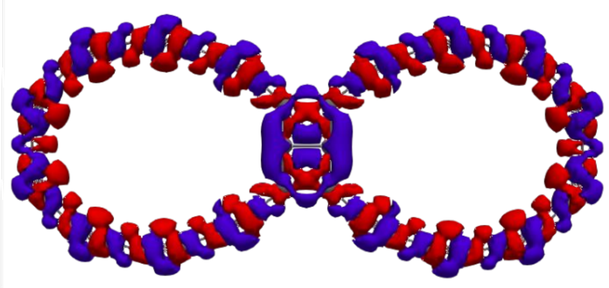</td></tr>
</table>

Table S6: Cartesian coordinates for optimized molecular structures of the singlet and triplet states of C_5-C_{29} optimized at the BHandHLYP and CASSCF levels of theory.

CASSCF C5 Singlet

6	1.972360873	-0.179469639	0.712058204
6	1.531648581	-0.005168686	2.090799607
6	1.271077805	0.623263790	-0.329808887
6	0.390075756	0.096864995	1.262060569
6	0.108142859	-0.228112053	-0.020381846

DFT/BHLYP C5 Singlet

6	-0.200206000	0.973940000	-0.426924000
6	0.939660000	0.385861000	0.326313000
6	-1.298188000	0.272191000	0.196160000
6	0.926094000	-0.955217000	-0.209868000
6	-0.370005000	-0.679652000	0.007608000

CASSCF C5 Triplet

6	2.110446963	-0.026913037	0.786803009
6	1.561159578	0.042026066	2.137031981
6	1.243040396	0.361795370	-0.207251174
6	0.522339720	-0.034780279	1.155010402
6	-0.163680784	-0.034749714	-0.156866570

DFT/BHLYP C5 Triplet

6	-0.260853000	0.958323000	-0.271647000
6	0.953955000	0.316386000	0.174654000
6	-1.403302000	0.313518000	0.119114000
6	1.020677000	-1.015982000	-0.131950000
6	-0.313122000	-0.575122000	0.003118000

CASSCF C7 Singlet

6	0.155902506	-0.018290614	0.061332871
6	-0.003063927	-0.017408885	1.316340768
6	1.061898326	0.001158198	2.198699512
6	0.876847974	-0.010461419	-1.145228473
6	2.295467594	0.019172247	1.895097555
6	2.092956832	0.009961040	-0.429019995
6	2.810730492	0.023648710	0.611147796

DFT/BHLYP C7 Singlet

6	-1.252720000	0.074214000	0.011737000
6	-1.159396000	1.393946000	0.011879000
6	0.230532000	1.274944000	-0.000508000
6	-0.966269000	-1.201801000	0.007437000
6	1.195374000	0.499189000	-0.010464000
6	1.610291000	-0.832743000	-0.015308000
6	0.341447000	-1.207364000	-0.004758000

CASSCF C7 Triplet

6	0.168780080	-0.018158804	0.035499084
6	0.026488128	-0.017004560	1.298473970
6	1.003844396	0.000415419	2.253426062
6	0.877015799	-0.010478537	-1.150683386

6	2.368788827	0.020331118	1.911008032
6	2.070946557	0.009579603	-0.447702267
6	2.774876011	0.023095038	0.608348538

DFT/BHLYP C7 Triplet

6	-1.355179000	0.006154000	0.005822000
6	-1.015630000	1.262415000	-0.152047000
6	0.291863000	1.275388000	0.067122000
6	-1.024102000	-1.251718000	0.158299000
6	1.412276000	0.676688000	0.151805000
6	1.407035000	-0.691703000	-0.159631000
6	0.281078000	-1.278757000	-0.071356000

CASSCF C9 Singlet

6	-0.564931809	0.017172796	1.622380071
6	1.489960029	0.032891801	0.803518189
6	-1.299156843	-0.016760537	-1.561247501
6	1.793813535	0.026163906	-0.392716872
6	0.017888610	-0.007038430	-2.109071945
6	1.172499780	0.008505365	-1.672406866
6	-1.628030971	0.000692518	0.971504739
6	-1.704897383	-0.011674467	-0.428503555
6	0.765110675	0.035216627	2.027036606

DFT/BHLYP C9 Singlet

6	-0.800452000	-1.491459000	-0.012430000
6	0.405994000	-1.956833000	-0.033481000
6	-1.846209000	-0.730731000	0.051515000
6	1.336924000	-1.071779000	0.065467000
6	1.933019000	0.070841000	-0.113595000
6	0.248076000	1.822080000	-0.166205000
6	1.360517000	1.190495000	0.139631000
6	-0.989355000	1.636351000	0.113624000
6	-1.648764000	0.531184000	-0.102344000

CASSCF C9 Triplet

6	-0.667635817	0.013452131	1.750114793
6	1.527764142	0.026275392	0.788420477
6	-1.218690652	-0.008045354	-1.481919163
6	1.835789329	0.021987567	-0.431207990
6	0.035172577	-0.002089984	-2.126661059
6	1.189841617	0.012574690	-1.668999980
6	-1.617225501	0.001997113	0.975007596
6	-1.773057382	-0.006813322	-0.420436116
6	0.730297308	0.025831345	1.876174309

DFT/BHLYP C9 Triplet

6	-0.826637000	-1.468180000	-0.002617000
6	0.423273000	-1.928626000	-0.005192000
6	-1.846195000	-0.740922000	-0.000809000
6	1.371352000	-1.094721000	-0.009093000
6	2.032168000	0.043301000	-0.012165000
6	0.230052000	1.679767000	-0.008644000
6	1.377260000	1.199143000	-0.011655000
6	-1.124473000	1.725856000	-0.004813000

6	-1.637051000	0.584531000	-0.002827000

CASSCF C11 Singlet

6	-1.728966085	-0.003688818	1.587302820
6	2.561547344	-0.024817018	0.247597185
6	-1.319553946	-0.002811884	-1.952489542
6	1.923469025	-0.020842137	-0.926801528
6	-0.102904155	-0.009911730	-2.157985159
6	1.263052651	-0.016399249	-1.989677107
6	-2.354900577	0.000834762	0.337744015
6	-2.196025641	0.000934974	-0.868936386
6	1.672256753	-0.021216876	1.263548507
6	-0.590855059	-0.009133964	2.060674579
6	0.788215291	-0.017029618	2.131722888

DFT/BHLYP C11 Singlet

6	-0.000291000	2.129746000	0.968991000
6	-0.000375000	2.076512000	-0.327643000
6	0.000495000	-1.175289000	1.785922000
6	-0.000089000	1.871207000	-1.578582000
6	0.000280000	0.627289000	-2.061750000
6	-0.000125000	-1.687448000	-1.427848000
6	0.000133000	-0.593112000	-2.225184000
6	-0.000169000	-2.146750000	-0.298041000
6	-0.000021000	-2.190099000	1.050431000
6	0.000196000	-0.006650000	2.381021000
6	-0.000036000	1.094594000	1.732687000

CASSCF C11 Triplet

6	-1.646087652	-0.003844266	1.306562466
6	2.723561178	-0.025764756	0.267426990
6	-1.300011896	-0.003151467	-1.648750989
6	2.036186549	-0.021430923	-0.882449662
6	-0.100537567	-0.009905728	-1.928516721
6	1.269974402	-0.016721649	-1.866097143
6	-2.661617072	0.002378189	0.361840275
6	-2.503173913	0.002695550	-0.969732742
6	1.794946914	-0.021663165	1.244842514
6	-0.529024573	-0.009568457	1.823316803
6	0.831119231	-0.017104886	2.024258481

CASPT2/svp C11 singlet

C	-1.895543973	-0.002954159	1.743267597
C	1.959896663	-0.021546020	0.061311622
C	-0.931148534	-0.004908693	-1.726878565
C	1.692838388	-0.019152546	-1.239401693
C	0.342769773	-0.011390056	-2.077684938
C	1.622593823	-0.017776306	-2.627966220
C	-2.287056358	0.000266418	0.473628412
C	-1.749217851	-0.001458354	-0.707901286
C	1.374705144	-0.019512902	1.224111475
C	-0.763029167	-0.009484997	2.382294348
C	0.548527686	-0.016163952	2.227919500

CASPT2/svp C11 triplet

C	-1.800528021	-0.003373078	1.654305157
C	2.267166239	-0.023269070	0.220094099
C	-1.210237998	-0.003484972	-1.923305027
C	1.976915962	-0.020719651	-1.049023876
C	0.005703271	-0.009460452	-2.322173720
C	1.368591436	-0.016599609	-2.228128653
C	-2.293439536	0.000236507	0.373790759
C	-2.054077901	0.000026197	-0.878296792
C	1.618026653	-0.020855198	1.380335444
C	-0.659484577	-0.009751533	2.217851889
C	0.696700352	-0.016830614	2.287251141

DFT/BHLYP C11 Triplet

6	-0.146877000	-2.215321000	0.010374000
6	1.186698000	-2.055531000	0.008583000
6	-2.183735000	0.434840000	0.001696000
6	1.926520000	-1.048517000	0.001809000
6	2.328136000	0.194424000	-0.004082000
6	0.799748000	2.204069000	-0.014474000
6	1.715359000	1.318713000	-0.008468000
6	-0.512999000	2.140778000	-0.010572000
6	-1.667461000	1.681512000	-0.004867000
6	-2.128727000	-0.779836000	0.008334000
6	-1.316663000	-1.870283000	0.011666000

CASSCF C13 Singlet

6	0.640831101	2.475039363	-0.007874049
6	1.781027286	2.023948256	-0.001433489
6	-2.350182691	-1.497282183	-0.001614123
6	2.512184810	0.835642879	0.007050304
6	2.739722946	-0.367850097	0.012392675
6	1.235178228	-2.399395465	0.014417661
6	2.156495183	-1.639668443	0.015201549
6	-0.115824370	-2.733387646	0.010693423
6	-1.252185659	-2.360431291	0.005771651
6	-2.574387677	-0.287756950	-0.006916597
6	-0.663054535	2.904579723	-0.015209082
6	-2.497115067	1.090673657	-0.013051974
6	-1.608237800	1.958937455	-0.012737931

DFT/BHLYP C13 Singlet

6	0.725925000	2.637277000	0.000000000
6	1.741891000	1.899061000	0.000000000
6	-2.187451000	-1.399926000	0.000000000
6	2.576335000	0.891903000	0.000000000
6	2.589289000	-0.345028000	0.000000000
6	1.202051000	-2.364714000	0.000000000
6	2.156679000	-1.594670000	0.000000000
6	-0.096862000	-2.631737000	0.000000000
6	-1.298706000	-2.372166000	0.000000000
6	-2.720362000	-0.275352000	0.000000000
6	-0.553239000	2.491904000	0.000000000

6	-2.370248000	0.976175000	0.000000000
6	-1.765302000	2.087272000	0.000000000

CASSCF C13 Triplet

6	0.684758620	2.235732176	-0.074698247
6	1.798022851	1.795790429	-0.074684580
6	-2.427545886	-1.552767907	-0.074781153
6	2.891006322	0.911400163	-0.074675189
6	2.750725633	-0.439469994	-0.074703566
6	1.057064613	-2.303576980	-0.074749709
6	2.244880152	-1.578050379	-0.074736793
6	-0.109933497	-2.564223462	-0.074759185
6	-1.462914273	-2.259240717	-0.074770120
6	-3.039221996	-0.297498782	-0.074783740
6	-0.700388398	2.291772718	-0.074722801
6	-2.834184773	0.914117807	-0.074772530
6	-1.820419336	1.873488696	-0.074747478

DFT/BHLYP C13 Triplet

6	0.710560000	2.554693000	0.000000000
6	1.828620000	1.994588000	0.000000000
6	-2.281918000	-1.457694000	0.000000000
6	2.521056000	0.861030000	0.000000000
6	2.677624000	-0.353104000	0.000000000
6	1.218684000	-2.379354000	0.000000000
6	2.138629000	-1.592975000	0.000000000
6	-0.104994000	-2.681658000	0.000000000
6	-1.270921000	-2.342708000	0.000000000
6	-2.645734000	-0.278154000	0.000000000
6	-0.583550000	2.627878000	0.000000000
6	-2.496166000	1.028333000	0.000000000
6	-1.711890000	2.019123000	0.000000000

CASSCF C15 Singlet

6	-1.134150948	2.812695723	0.003528867
6	-2.191483445	1.919929404	0.002295737
6	3.006467502	-0.186362477	0.001637885
6	-2.672847650	0.814727154	0.000742240
6	-3.040483441	-0.498401340	-0.001309135
6	-2.064895197	-2.881998057	-0.003960530
6	-2.583362301	-1.647169014	-0.002647139
6	-0.732093648	-2.989166892	-0.003281150
6	0.503791501	-3.068765364	-0.002689210
6	3.013685470	1.038315245	0.002383837
6	0.020801060	3.219882751	0.003557221
6	2.351086196	2.249461420	0.002903267
6	1.385053481	2.984332573	0.003284574
6	2.515595123	-1.479891645	0.000169911
6	1.624166297	-2.292386481	-0.001138376

DFT/BHLYP C15 Singlet

6	-1.216102000	2.724613000	0.005118000
6	-2.369623000	2.065522000	0.002647000
6	2.990293000	-0.277789000	0.001131000
6	-2.796820000	0.898712000	0.000215000

6	-3.132121000	-0.365898000	-0.003156000
6	-1.845157000	-2.566919000	-0.004428000
6	-2.516794000	-1.473419000	-0.004181000
6	-0.586388000	-2.865921000	0.000910000
6	0.650501000	-3.088302000	-0.002472000
6	2.943414000	0.939753000	0.001303000
6	-0.055675000	3.113108000	0.004285000
6	2.241759000	2.084363000	0.002347000
6	1.240811000	2.772776000	0.003939000
6	2.702380000	-1.581046000	-0.000980000
6	1.759178000	-2.376031000	-0.001198000

CASSCF C15 Triplet

6	-1.281507462	2.777331418	0.002799168
6	-2.315268156	2.125861179	0.001482498
6	3.133134700	-0.254586272	0.000871067
6	-2.909449555	0.872396407	0.000008307
6	-3.061095100	-0.314810392	-0.001053387
6	-1.866376722	-2.541617531	-0.002585884
6	-2.648962720	-1.635141574	-0.002029864
6	-0.600113555	-3.098518834	-0.002865234
6	0.596550503	-3.050522354	-0.002695937
6	2.911841014	0.947385435	0.002352746
6	0.004635634	3.210160939	0.004043550
6	2.365071808	2.189277970	0.003781727
6	1.212779189	2.768393317	0.004045807
6	2.613007433	-1.540311581	-0.000768329
6	1.847082990	-2.460095127	-0.001908235

CASSCF C15_2 Triplet

6	-1.126048676	2.861793433	0.002984408
6	-2.108277794	1.882631104	0.001558504
6	3.072447389	-0.154570135	0.001212685
6	-2.671926095	0.827859545	0.000240322
6	-2.880037228	-0.537832272	-0.001232548
6	-2.161121971	-3.008039721	-0.003416139
6	-2.731256768	-1.727129323	-0.002348721
6	-0.765179479	-3.139020169	-0.003164779
6	0.409469711	-2.900741057	-0.002619060
6	3.020540813	1.056807927	0.002577465
6	0.038475250	3.199389978	0.003804379
6	2.402035142	2.292087710	0.003740743
6	1.406701679	3.007953078	0.004135248
6	2.458021953	-1.397990453	-0.000395409
6	1.637486074	-2.267996644	-0.001599099

DFT/BHLYP C15 Triplet

6	-1.253856000	2.743911000	0.004566000
6	-2.320121000	2.079940000	0.003325000
6	3.105281000	-0.276189000	0.001999000
6	-2.902304000	0.899704000	0.001563000
6	-3.100955000	-0.316121000	-0.001125000
6	-1.831637000	-2.488840000	-0.004277000
6	-2.622662000	-1.566423000	-0.003331000
6	-0.585276000	-3.010105000	-0.003760000

6	0.624426000	-2.998301000	-0.002428000
6	2.887872000	0.939920000	0.002317000
6	-0.018523000	3.110206000	0.001774000
6	2.296790000	2.111084000	0.001196000
6	1.216988000	2.761436000	0.003511000
6	2.640035000	-1.528146000	0.000702000
6	1.865272000	-2.466873000	-0.000554000

CASSCF C17 Singlet

6	-1.127037958	-0.021479284	-3.341293170
6	-2.191980940	-0.022557746	-2.485294867
6	-3.010174239	-0.022907943	-1.573829376
6	-3.341036266	-0.023696659	-0.225070254
6	-3.322436502	-0.024223676	0.969606240
6	-2.788197664	-0.024465697	2.246448292
6	-1.944026746	-0.024441971	3.093772338
6	-0.669230437	-0.023330297	3.631412753
6	0.524980370	-0.023434434	3.550229505
6	1.791416512	-0.023485855	2.998111565
6	2.563604414	-0.023487042	2.082737856
6	3.150470975	-0.022643878	0.834172632
6	3.203625133	-0.022296928	-0.362547370
6	3.045594118	-0.021351556	-1.737881707
6	2.266383844	-0.020749654	-2.697700846
6	1.425249997	-0.019548394	-3.784897069
6	0.094876079	-0.020651648	-3.524696991

DFT/BHLYP C17 Singlet

6	-1.218535000	0.000000000	-3.173517000
6	-2.369679000	0.000000000	-2.591251000
6	-3.035703000	0.000000000	-1.538733000
6	-3.484215000	0.000000000	-0.307241000
6	-3.298538000	0.000000000	0.908515000
6	-2.768622000	0.000000000	2.122985000
6	-1.841835000	0.000000000	2.917436000
6	-0.608852000	0.000000000	3.421668000
6	0.608852000	0.000000000	3.421668000
6	1.841835000	0.000000000	2.917436000
6	2.768622000	0.000000000	2.122985000
6	3.298538000	0.000000000	0.908515000
6	3.484215000	0.000000000	-0.307241000
6	3.035703000	0.000000000	-1.538733000
6	2.369679000	0.000000000	-2.591251000
6	1.218535000	0.000000000	-3.173517000
6	0.000000000	0.000000000	-3.519725000

CASSCF C17 Triplet

6	-1.149824570	-0.000000140	-3.090589968
6	-2.383197320	-0.000000058	-2.466512080
6	-3.215329326	0.000000062	-1.569946242
6	-3.553718523	0.000000129	-0.222319816
6	-3.486745617	0.000000111	0.971072347
6	-2.851753956	0.000000082	2.198903389
6	-1.977393897	-0.000000021	3.016258951
6	-0.663197055	0.000000048	3.441903929

6	0.533242655	0.000000160	3.405620907
6	1.786719147	0.000000267	2.825248867
6	2.662564124	0.000000435	2.010157242
6	3.310248486	0.000000430	0.789505187
6	3.553785336	0.000000192	-0.382269522
6	3.524654677	-0.000000341	-1.778513255
6	2.475553872	-0.000000268	-2.599188235
6	1.405122379	0.000000017	-3.252292601
6	0.029269591	-0.000000106	-3.297042099

DFT/BHLYP C17 Triplet

6	-1.256094000	0.000001000	-3.259313000
6	-2.337823000	0.000001000	-2.545437000
6	-3.120169000	0.000000000	-1.575121000
6	-3.454404000	-0.000001000	-0.298208000
6	-3.368968000	-0.000001000	0.925658000
6	-2.753726000	-0.000001000	2.114558000
6	-1.865567000	0.000000000	2.941678000
6	-0.604475000	0.000001000	3.421085000
6	0.604475000	0.000001000	3.421085000
6	1.865567000	0.000001000	2.941678000
6	2.753726000	0.000001000	2.114558000
6	3.368968000	0.000000000	0.925658000
6	3.454404000	-0.000001000	-0.298208000
6	3.120169000	-0.000001000	-1.575121000
6	2.337823000	-0.000001000	-2.545437000
6	1.256094000	0.000000000	-3.259313000
6	0.000000000	0.000001000	-3.449803000

CASSCF C19 Singlet

6	-2.119041264	0.124864181	2.979193270
6	-2.970599374	0.178043105	1.896457855
6	-3.390915653	0.205162376	0.771262932
6	-3.718592214	0.227118812	-0.553037130
6	-3.489741551	0.215043638	-1.753460371
6	-3.238270129	0.201855359	-3.079694000
6	-2.011318667	0.127741693	-3.563148728
6	-0.869005928	0.058768889	-4.058295608
6	0.444075009	-0.022034359	-3.739202010
6	1.589627057	-0.092786659	-3.354111432
6	2.592597569	-0.155665267	-2.423057660
6	3.318666475	-0.201436243	-1.469287121
6	3.709667444	-0.227002582	-0.147345183
6	3.765666838	-0.231758111	1.046155966
6	3.393851437	-0.210495578	2.384118547
6	2.598977726	-0.162953462	3.310817571
6	1.383378958	-0.089514886	3.967459067
6	0.167309782	-0.015472136	4.062725735
6	-1.165383515	0.065998230	3.722450301

DFT/BHLYP C19 Singlet

6	-2.294674000	0.138127000	3.017671000
6	-3.240675000	0.198778000	2.204055000
6	-3.605073000	0.220595000	0.939169000
6	-3.825295000	0.233468000	-0.269346000

6	-3.426323000	0.207196000	-1.538621000
6	-2.854272000	0.174373000	-2.612876000
6	-1.791249000	0.109899000	-3.424380000
6	-0.667843000	0.043377000	-3.875657000
6	0.672036000	-0.037184000	-3.877604000
6	1.811070000	-0.105440000	-3.470841000
6	2.870396000	-0.169853000	-2.650365000
6	3.463306000	-0.206784000	-1.591626000
6	3.827318000	-0.230352000	-0.306205000
6	3.617691000	-0.221784000	0.896797000
6	3.199942000	-0.198140000	2.154726000
6	2.280423000	-0.148936000	2.978729000
6	1.250236000	-0.081926000	3.782686000
6	-0.008878000	-0.006741000	3.816193000
6	-1.287184000	0.076804000	3.827497000

CASSCF C19 Triplet

6	-2.373433693	0.140289784	3.096999370
6	-3.232115462	0.193140624	2.230949537
6	-3.666903208	0.221420488	0.918353897
6	-3.778013343	0.230121598	-0.272355636
6	-3.457544490	0.212967674	-1.614710305
6	-2.869268249	0.178899097	-2.655983173
6	-1.809580655	0.115613737	-3.538630013
6	-0.693978544	0.048024579	-3.966072292
6	0.684494957	-0.036338547	-3.970599325
6	1.800715554	-0.105306575	-3.544918042
6	2.863980461	-0.171836492	-2.666907085
6	3.453146791	-0.209598780	-1.626208040
6	3.777471016	-0.231408745	-0.284866351
6	3.668577639	-0.226188346	0.906089706
6	3.236304804	-0.201019314	2.219646626
6	2.378485248	-0.149120690	3.086484848
6	1.284192604	-0.082691838	3.874718731
6	0.003492968	-0.004751356	3.923026335
6	-1.279074399	0.073260104	3.884983214

CASSCF C19_2 Triplet

6	-2.156664677	0.127432430	2.971748464
6	-2.970967882	0.178731438	1.853806569
6	-3.461890289	0.210200692	0.765024128
6	-3.711670106	0.227246511	-0.592303745
6	-3.691230404	0.227530009	-1.789821577
6	-3.387681279	0.210713331	-3.153400003
6	-2.080028891	0.131471927	-3.637650557
6	-0.894857661	0.059316267	-3.796422975
6	0.476049945	-0.024513603	-3.668120518
6	1.583595893	-0.092654948	-3.221986503
6	2.696087741	-0.161570362	-2.409695871
6	3.384946393	-0.204870566	-1.432855800
6	3.848112173	-0.234864380	-0.133164435
6	3.840311870	-0.236029477	1.062050216
6	3.455023536	-0.214381099	2.393015669
6	2.621133048	-0.164748647	3.269412987
6	1.399897701	-0.091116989	3.902441421

6	0.182434411	-0.016906972	3.991685325
6	-1.141651522	0.064491437	3.626239205

DFT/BHLYP C19 Triplet

6	-2.361748000	0.139218000	3.033236000
6	-3.251694000	0.197881000	2.151852000
6	-3.736736000	0.227469000	0.936043000
6	-3.886216000	0.240180000	-0.289383000
6	-3.525341000	0.218166000	-1.567733000
6	-2.886520000	0.178298000	-2.605831000
6	-1.816091000	0.112743000	-3.413189000
6	-0.678939000	0.043277000	-3.822354000
6	0.667448000	-0.037763000	-3.821097000
6	1.808049000	-0.106419000	-3.427236000
6	2.878724000	-0.170945000	-2.613081000
6	3.522462000	-0.209529000	-1.584185000
6	3.879974000	-0.234478000	-0.297039000
6	3.740021000	-0.228218000	0.921512000
6	3.251904000	-0.202649000	2.146813000
6	2.374687000	-0.152826000	3.027472000
6	1.260538000	-0.084418000	3.690575000
6	0.007249000	-0.007382000	3.843136000
6	-1.256821000	0.072872000	3.690491000

CASSCF C21 Singlet

6	2.247130742	-3.214224626	-0.445381237
6	2.112415453	-3.020549847	-1.618300822
6	1.865394308	-2.668027395	-2.931377033
6	1.362814690	-1.950241765	-3.799569205
6	-0.613510924	0.877266099	-4.274876910
6	-1.247650936	1.786515712	-3.483938824
6	-1.769069740	2.533744303	-2.668292458
6	-2.115970280	3.027364320	-1.422217309
6	-2.304557612	3.292322624	-0.272748649
6	-2.294167150	3.273071967	1.107225580
6	-2.117590646	3.019592850	2.262281605
6	-1.672284743	2.385063307	3.404593467
6	-1.126386432	1.608820608	4.132464616
6	-0.372291726	0.536551417	4.565900597
6	0.313829199	-0.441077302	4.499365305
6	1.055379688	-1.500867803	4.019494625
6	1.565934675	-2.233797384	3.223685719
6	2.007063694	-2.868271225	2.082208394
6	2.187006275	-3.127846274	0.928414180
6	0.081627345	-0.118833421	-4.488182879
6	0.834884120	-1.196577163	-4.820751762

DFT/BHLYP C21 Singlet

6	2.410678000	-3.447822000	-0.348704000
6	2.281403000	-3.271679000	-1.555782000
6	1.873482000	-2.694356000	-2.660900000
6	1.383975000	-1.993621000	-3.562348000
6	-0.703527000	1.015291000	-4.056041000
6	-1.383975000	1.993621000	-3.562348000
6	-1.873482000	2.694356000	-2.660900000

6	-2.281403000	3.271679000	-1.555782000
6	-2.410678000	3.447822000	-0.348704000
6	-2.388529000	3.405661000	0.977362000
6	-2.122226000	3.017895000	2.100612000
6	-1.664006000	2.360718000	3.168886000
6	-1.084840000	1.536546000	3.845604000
6	-0.349822000	0.495020000	4.254984000
6	0.349822000	-0.495020000	4.254984000
6	1.084840000	-1.536546000	3.845604000
6	1.664006000	-2.360718000	3.168886000
6	2.122226000	-3.017895000	2.100612000
6	2.388529000	-3.405661000	0.977362000
6	0.000000000	0.000000000	-4.327349000
6	0.703527000	-1.015291000	-4.056041000

CASSCF C21 Triplet

6	2.340467799	-3.344351798	-0.426171884
6	2.227560741	-3.182951388	-1.604963619
6	1.955033598	-2.793442942	-2.903809228
6	1.421725259	-2.031652163	-3.740064550
6	-0.619284110	0.885002627	-4.109070017
6	-1.322971065	1.890506726	-3.489398915
6	-1.850431524	2.644040283	-2.685817835
6	-2.213754774	3.163216199	-1.453278523
6	-2.390598151	3.416010131	-0.299510370
6	-2.356026019	3.366621445	1.079417043
6	-2.155580419	3.080100001	2.223056627
6	-1.681619300	2.402685992	3.327121058
6	-1.135106354	1.621673216	4.050037508
6	-0.375371984	0.536207806	4.433677869
6	0.310697163	-0.443859190	4.424477406
6	1.049665241	-1.499581151	3.931941964
6	1.596694826	-2.281239982	3.210517405
6	2.047397950	-2.925404496	2.076613595
6	2.276215159	-3.252527432	0.949611211
6	0.046201322	-0.066054436	-4.415039784
6	0.829084644	-1.185000449	-4.579349961

DFT/BHLYP C21 Triplet

6	2.454662000	-3.507302000	-0.347301000
6	2.281254000	-3.261917000	-1.543593000
6	1.916738000	-2.743667000	-2.689757000
6	1.380472000	-1.975177000	-3.517723000
6	-0.720895000	1.033207000	-4.102455000
6	-1.380460000	1.975159000	-3.517702000
6	-1.916725000	2.743649000	-2.689734000
6	-2.281246000	3.261905000	-1.543574000
6	-2.454660000	3.507299000	-0.347285000
6	-2.386927000	3.409437000	0.974257000
6	-2.142708000	3.057561000	2.116586000
6	-1.657566000	2.364980000	3.155923000
6	-1.086389000	1.549664000	3.845975000
6	-0.347017000	0.494506000	4.238556000
6	0.347008000	-0.494493000	4.238550000
6	1.086378000	-1.549648000	3.845957000

6	1.657553000	-2.364962000	3.155901000
6	2.142697000	-3.057545000	2.116565000
6	2.386920000	-3.409429000	0.974241000
6	0.000005000	-0.000006000	-4.260921000
6	0.720906000	-1.033222000	-4.102469000

CASSCF C23 Singlet

6	0.268544546	-4.790873707	0.008348685
6	-1.112046480	-4.767612205	0.009520329
6	-2.253746312	-4.418891755	0.012269295
6	-3.439629116	-3.699244119	0.017406238
6	-4.642398524	-0.196430090	0.030731388
6	-4.339940181	1.144122885	0.030877735
6	-3.712627525	2.190400358	0.028314523
6	-2.787788212	3.214810879	0.023578356
6	-1.809476603	3.908709320	0.018071513
6	1.890707446	5.106772744	-0.004912136
6	2.815955075	4.142500531	-0.006660787
6	3.707140133	3.291565665	-0.007700070
6	4.159469897	2.013008241	-0.005387276
6	4.463507856	0.846197720	-0.003135309
6	4.369744183	-0.520892135	0.000578094
6	4.104464059	-1.689627464	0.003673285
6	3.409653214	-2.877023116	0.006635128
6	2.609182681	-3.766191970	0.007945763
6	1.416223040	-4.456459622	0.008385030
6	-4.605007937	-1.405987968	0.028261585
6	-4.137963450	-2.698173550	0.023176538
6	-0.629247875	4.593804101	0.010262529
6	0.569569611	4.820408212	0.002990004

DFT/BHLYP C23 Singlet

6	0.384534000	-4.547728000	0.000505000
6	-0.950048000	-4.466565000	0.002181000
6	-2.070017000	-3.989966000	-0.000205000
6	-3.208765000	-3.306287000	0.001917000
6	-4.698715000	0.042752000	-0.001206000
6	-4.748984000	1.328960000	0.007733000
6	-4.040313000	2.402985000	0.002526000
6	-3.340215000	3.440952000	0.007660000
6	-2.164868000	4.024923000	0.002504000
6	1.504338000	4.311413000	-0.001336000
6	2.686948000	3.687793000	-0.004567000
6	3.627777000	2.923651000	-0.003682000
6	4.348108000	1.792016000	-0.004885000
6	4.728417000	0.643474000	-0.004190000
6	4.744718000	-0.699701000	-0.003751000
6	4.419441000	-1.864403000	-0.002928000
6	3.709086000	-3.004505000	-0.001441000
6	2.788301000	-3.789295000	-0.000895000
6	1.585283000	-4.382883000	0.000887000
6	-4.638459000	-1.207401000	0.003365000
6	-3.937542000	-2.317350000	-0.003382000
6	-1.028511000	4.492268000	0.003218000
6	0.299488000	4.484897000	-0.002023000

CASSCF C23 Triplet

6	0.280904857	-4.795294977	0.014103232
6	-1.099588188	-4.766957800	0.019483429
6	-2.235956030	-4.400651561	0.021733068
6	-3.421893453	-3.682422123	0.023310493
6	-4.619714640	-0.195473871	0.025623643
6	-4.307911988	1.149596823	0.024966549
6	-3.741643641	2.214960427	0.022647633
6	-2.793537378	3.219910915	0.018189067
6	-1.848415857	3.950196668	0.014431299
6	1.891168330	5.183939320	0.002542572
6	2.875807979	4.198479547	0.000199525
6	3.576603235	3.227224453	-0.001275552
6	4.153387446	1.975609322	-0.003040595
6	4.419022608	0.809942090	-0.004198691
6	4.399006223	-0.567767772	-0.004735116
6	4.126535367	-1.731952524	-0.004582558
6	3.448687489	-2.932172242	-0.002218785
6	2.632414784	-3.805743877	0.001615489
6	1.438102617	-4.495965871	0.007793698
6	-4.615845822	-1.403852426	0.025362694
6	-4.139769095	-2.695686474	0.024166560
6	-0.620423453	4.577872981	0.010380489
6	0.517348135	4.951101925	0.006732297

DFT/BHLYP C23 Triplet

6	0.344922000	-4.658652000	0.010204000
6	-0.982616000	-4.590371000	0.010974000
6	-2.132684000	-4.156113000	0.009037000
6	-3.223439000	-3.422645000	0.003964000
6	-4.666212000	-0.008507000	-0.006608000
6	-4.526378000	1.262340000	-0.006066000
6	-3.982035000	2.420307000	-0.004320000
6	-3.214639000	3.419834000	-0.001491000
6	-2.136316000	4.142346000	0.004047000
6	1.545115000	4.435578000	0.009904000
6	2.717794000	3.808179000	0.006534000
6	3.621295000	2.992276000	0.000655000
6	4.308433000	1.840967000	-0.007104000
6	4.644839000	0.678481000	-0.012735000
6	4.640656000	-0.668042000	-0.015319000
6	4.313938000	-1.830821000	-0.013806000
6	3.617246000	-2.982925000	-0.008106000
6	2.725121000	-3.801026000	-0.001225000
6	1.540875000	-4.428756000	0.006088000
6	-4.531494000	-1.262400000	-0.004292000
6	-3.984056000	-2.438270000	0.001136000
6	-0.976974000	4.591103000	0.006439000
6	0.336610000	4.657116000	0.010092000

CASSCF C25 Singlet

6	5.844652700	1.464164916	-0.016699663
6	4.875470331	2.438750429	-0.016699295
6	1.705251904	4.377661842	-0.006811007

6	0.341556934	4.570787451	-0.001472786
6	-3.267006796	3.698195299	0.005975887
6	-4.296978856	2.782899453	0.001696636
6	-4.992500663	1.810281129	-0.004769753
6	-5.409997966	0.496424617	-0.014130596
6	-5.471762525	-0.697676948	-0.022613503
6	-3.474606490	-3.983430875	-0.037342165
6	-2.393985302	-4.494854260	-0.035089545
6	-1.049337240	-4.797258272	-0.030617197
6	0.144422982	-4.739544804	-0.025676725
6	1.486636242	-4.425049426	-0.020399446
6	2.561682440	-3.905210962	-0.016542139
6	3.712810770	-3.139534210	-0.013298388
6	4.467104558	-2.178566467	-0.012419463
6	5.216401742	-1.044440256	-0.012904367
6	5.458371274	0.163154930	-0.014569718
6	-2.187136386	4.211360388	0.005898444
6	-0.853905963	4.557818646	0.003076697
6	-5.116144866	-2.030466377	-0.030984997
6	-4.487238158	-3.047319229	-0.035790713
6	2.829355212	3.969979882	-0.011178871
6	4.075700584	3.376122022	-0.015131269

DFT/BHLYP C25 Singlet

6	5.188689000	1.305807000	0.004920000
6	4.512593000	2.409564000	-0.001807000
6	1.626837000	4.702721000	0.000266000
6	0.306176000	4.880389000	0.000472000
6	-3.241648000	3.864713000	-0.000609000
6	-4.188456000	2.911131000	-0.001558000
6	-4.818122000	1.879874000	-0.002388000
6	-5.177082000	0.583368000	-0.003234000
6	-5.188949000	-0.624360000	-0.003963000
6	-3.282102000	-3.918932000	-0.004431000
6	-2.225954000	-4.507166000	-0.004376000
6	-0.940512000	-4.893761000	-0.003119000
6	0.270927000	-4.900748000	-0.002164000
6	1.589066000	-4.676999000	0.000241000
6	2.670780000	-4.118225000	0.002622000
6	3.766280000	-3.370543000	0.005289000
6	4.457848000	-2.352076000	0.007407000
6	5.127799000	-1.229074000	0.008945000
6	5.162409000	0.028952000	0.008470000
6	-2.183721000	4.451750000	0.000122000
6	-0.907494000	4.860814000	0.000609000
6	-4.844444000	-1.925048000	-0.004304000
6	-4.229797000	-2.964929000	-0.004788000
6	2.718420000	4.151360000	-0.002215000
6	3.835455000	3.451416000	-0.000406000

CASSCF C25 Triplet

6	5.523667989	1.423701558	-0.011091024
6	4.759752068	2.509318634	-0.005018075
6	1.690360627	4.588297120	0.005230865
6	0.325488646	4.782813453	0.001525276

6	-3.248128111	3.784278144	-0.013933246
6	-4.245072547	2.832702798	-0.015643437
6	-4.906566526	1.836683272	-0.015599679
6	-5.301959449	0.515731187	-0.014583432
6	-5.344614033	-0.679167262	-0.013497539
6	-3.410020311	-4.011097965	-0.022329208
6	-2.350979355	-4.566017634	-0.027471553
6	-1.020143313	-4.924819432	-0.031814879
6	0.175052180	-4.917974363	-0.033405463
6	1.525322113	-4.636029903	-0.032816316
6	2.604557861	-4.125801437	-0.029856395
6	3.734183870	-3.325431329	-0.025247608
6	4.460998707	-2.345156734	-0.021057253
6	5.030032201	-1.096115964	-0.017301081
6	5.329376468	0.066876226	-0.014569304
6	-2.191078050	4.342816658	-0.010283341
6	-0.868596322	4.730040120	-0.004267740
6	-5.005705848	-2.016179298	-0.014204117
6	-4.389629662	-3.040920665	-0.017087074
6	2.813888480	4.181216838	0.004511338
6	4.028628778	3.524484899	0.001316344

DFT/BHLYP C25 Triplet

6	4.913141000	1.270572000	0.008791000
6	4.427194000	2.452570000	0.006247000
6	1.590836000	4.859804000	-0.003621000
6	0.299220000	5.085722000	-0.005382000
6	-3.220434000	3.948261000	-0.003070000
6	-4.125924000	2.965390000	0.000277000
6	-4.716932000	1.906784000	0.002491000
6	-5.044432000	0.603355000	0.002470000
6	-5.045423000	-0.605120000	0.002003000
6	-3.206062000	-3.949041000	-0.006949000
6	-2.179630000	-4.594397000	-0.007481000
6	-0.913561000	-5.022913000	-0.007139000
6	0.305075000	-5.090073000	-0.004980000
6	1.610052000	-4.858175000	-0.001607000
6	2.714185000	-4.312195000	0.001551000
6	3.730026000	-3.483229000	0.004860000
6	4.433098000	-2.455076000	0.007096000
6	4.919314000	-1.254888000	0.008774000
6	5.048084000	-0.000286000	0.009001000
6	-2.184803000	4.594047000	-0.004862000
6	-0.930749000	5.022448000	-0.007056000
6	-4.707638000	-1.908404000	-0.001645000
6	-4.121771000	-2.965170000	-0.003583000
6	2.706600000	4.306595000	-0.000064000
6	3.713853000	3.492658000	0.003878000

CASSCF C27 Singlet

6	2.520417690	4.822851518	0.000473929
6	3.719275017	4.155949358	0.000161293
6	5.668507429	1.005745865	-0.000256419
6	5.748787809	-0.372068238	-0.000268952
6	4.188015182	-3.717514625	-0.000531431

6	3.096886733	-4.558591269	-0.000723222
6	1.987489975	-5.004308347	-0.000937534
6	0.633796076	-5.256034925	-0.001223996
6	-0.558784807	-5.166523661	-0.001463705
6	-4.059148509	-3.462091734	-0.001545949
6	-4.825471921	-2.533865130	-0.001302523
6	-6.277103945	1.053552250	0.000213889
6	-5.393463748	2.075456760	0.000647937
6	-4.636659611	3.031610440	0.000985100
6	-3.524363101	3.820824206	0.001193149
6	-2.496578815	4.438208468	0.001289173
6	-1.211271111	4.938630702	0.001245825
6	-0.019860991	5.196304618	0.001054038
6	1.350767204	5.130078453	0.000751843
6	4.957461599	-2.802715942	-0.000401794
6	5.523118942	-1.545488846	-0.000300701
6	-1.892535722	-4.830639686	-0.001642750
6	-2.947639133	-4.264226563	-0.001654493
6	5.309713592	2.144016890	-0.000222588
6	4.640231378	3.356950877	-0.000087846
6	-5.607904870	-1.424792781	-0.000888865
6	-5.893687343	-0.231320659	-0.000381412

DFT/BHLYP C27 Singlet

6	2.464127000	5.130899000	-0.002312000
6	3.424621000	4.242342000	-0.006598000
6	5.215420000	0.999641000	0.002146000
6	5.262359000	-0.335205000	0.003771000
6	3.948368000	-3.792115000	0.001411000
6	2.949471000	-4.689729000	-0.000345000
6	1.893687000	-5.277673000	-0.002229000
6	0.592140000	-5.612291000	-0.003653000
6	-0.615987000	-5.620906000	-0.004505000
6	-3.984690000	-3.827920000	-0.002711000
6	-4.649887000	-2.818355000	-0.000836000
6	-5.213679000	0.967178000	0.002126000
6	-4.822775000	2.117756000	0.001651000
6	-4.240314000	3.313012000	0.002888000
6	-3.386737000	4.191465000	0.002426000
6	-2.404943000	5.066087000	0.003470000
6	-1.216130000	5.436851000	0.001223000
6	0.020532000	5.807021000	0.001637000
6	1.254068000	5.464817000	-0.001812000
6	4.611016000	-2.780374000	0.003327000
6	5.115011000	-1.538064000	0.003562000
6	-1.920901000	-5.299225000	-0.004504000
6	-2.982894000	-4.722887000	-0.003816000
6	4.834488000	2.155968000	-0.001295000
6	4.283055000	3.361608000	-0.002337000
6	-5.144557000	-1.571295000	0.000091000
6	-5.284870000	-0.368605000	0.001405000

CASSCF C27 Triplet

6	2.548512282	4.789337433	0.103303919
6	3.693572263	4.009850080	0.091826244

6	5.676106405	0.876877139	0.048811367
6	5.831554856	-0.494858722	0.030114993
6	4.412274017	-3.907813977	-0.021358323
6	3.242946424	-4.650727150	-0.033873498
6	2.110330625	-5.026892168	-0.040198876
6	0.741921532	-5.200488800	-0.042203259
6	-0.445263651	-5.066661825	-0.038468009
6	-3.901417418	-3.256699666	-0.010750975
6	-4.707525661	-2.374601757	-0.000382302
6	-6.327952160	1.216016366	0.045472556
6	-5.491455730	2.328037986	0.061852421
6	-4.653775033	3.183433146	0.074226311
6	-3.555498383	4.016186401	0.086456630
6	-2.494097408	4.564312891	0.095012943
6	-1.184139130	4.995430370	0.102883955
6	-0.004524888	5.176396269	0.107364050
6	1.379091866	5.127749589	0.108058501
6	5.204754987	-2.982442289	-0.006263294
6	5.671651790	-1.677334277	0.013272793
6	-1.765991755	-4.674661069	-0.030862004
6	-2.816557842	-4.105199467	-0.021967317
6	5.273339787	2.001226189	0.063794537
6	4.544389204	3.173198852	0.079821594
6	-5.448237513	-1.212893188	0.013571791
6	-5.933690154	-0.118398545	0.027565898

DFT/BHLYP C27 Triplet

6	2.460174000	4.917118000	0.002111000
6	3.526071000	4.190166000	0.002420000
6	5.412986000	0.979989000	0.000630000
6	5.474272000	-0.337922000	-0.000611000
6	4.024446000	-3.747415000	-0.002959000
6	2.992997000	-4.602742000	-0.003156000
6	1.914757000	-5.151977000	-0.002799000
6	0.605257000	-5.459111000	-0.002011000
6	-0.602301000	-5.460448000	-0.001041000
6	-4.023065000	-3.747752000	0.001229000
6	-4.739654000	-2.768616000	0.001130000
6	-5.412805000	0.980703000	-0.000703000
6	-5.040673000	2.154862000	-0.001046000
6	-4.400799000	3.299099000	-0.001345000
6	-3.530245000	4.188369000	-0.001279000
6	-2.459186000	4.918305000	-0.000957000
6	-1.268046000	5.325725000	-0.000236000
6	0.000704000	5.498181000	0.000753000
6	1.263475000	5.326185000	0.001841000
6	4.740635000	-2.764689000	-0.002506000
6	5.278785000	-1.547174000	-0.001678000
6	-1.912325000	-5.151831000	0.000053000
6	-2.990522000	-4.605557000	0.000776000
6	5.037637000	2.158055000	0.002187000
6	4.401018000	3.297683000	0.002659000
6	-5.278277000	-1.547197000	0.000650000
6	-5.475321000	-0.342011000	0.000070000

CASSCF C29 Singlet

6	1.597990673	5.022372662	-0.001322703
6	0.279696103	5.408145154	-0.001373455
6	-3.455284988	5.283844215	-0.001579145
6	-4.773760122	5.002732665	-0.001653341
6	-5.808926838	1.266197094	-0.001167928
6	-5.784405999	0.063518381	-0.000782206
6	-5.443580980	-1.264425984	-0.000341751
6	-5.004889427	-2.378245241	0.000128901
6	-4.234423122	-3.517549969	0.000758521
6	-1.177453276	-5.633142361	0.001956899
6	0.181214884	-5.859850605	0.002107144
6	3.757085541	-4.889622506	0.001679546
6	4.801842227	-3.988117585	0.001186981
6	5.503954659	-3.023154493	0.000715185
6	6.098562879	-1.772092580	0.000166494
6	6.249260667	-0.562618489	-0.000483386
6	6.078184161	0.807024280	-0.000967850
6	5.560768197	1.911376501	-0.001208151
6	4.773021063	3.033234566	-0.001336555
6	-5.734969479	2.622216805	-0.001477283
6	-5.245756814	3.745692628	-0.001570251
6	-3.418091806	-4.391114882	0.001234271
6	-2.273470519	-5.156162121	0.001638529
6	-2.273734860	5.592725437	-0.001518078
6	-0.913968866	5.540853495	-0.001415374
6	1.375867695	-5.826172188	0.002036192
6	2.701961502	-5.450046509	0.001888385
6	2.702748542	4.565949054	-0.001283885
6	3.880556305	3.846437577	-0.001303709

DFT/BHLYP C29 Singlet

6	1.938170000	5.378410000	-0.003624000
6	0.754475000	5.662225000	-0.004247000
6	-3.004401000	4.888038000	-0.002489000
6	-4.009579000	4.216321000	-0.001792000
6	-5.951307000	0.914020000	0.001408000
6	-6.053206000	-0.289234000	0.001453000
6	-5.851454000	-1.618795000	0.001927000
6	-5.400170000	-2.738791000	0.001347000
6	-4.629264000	-3.840525000	0.001349000
6	-1.390169000	-5.628346000	0.000582000
6	-0.052842000	-5.743623000	0.000560000
6	3.446727000	-4.507678000	0.000944000
6	4.478377000	-3.667283000	0.000633000
6	5.167732000	-2.656590000	-0.002418000
6	5.826144000	-1.517353000	0.001005000
6	5.992237000	-0.285406000	0.001827000
6	6.144634000	0.997178000	0.003315000
6	5.641169000	2.170973000	0.001897000
6	5.146916000	3.325973000	0.000865000
6	-5.545534000	2.195746000	0.000321000
6	-4.922005000	3.230312000	-0.000268000
6	-3.724584000	-4.640615000	0.000795000
6	-2.552720000	-5.298636000	0.000518000

6	-1.774689000	5.422962000	-0.003805000
6	-0.580923000	5.629179000	-0.003932000
6	1.143612000	-5.562380000	0.000629000
6	2.417726000	-5.152166000	0.000969000
6	3.187662000	4.936588000	-0.002672000
6	4.157264000	4.179501000	-0.002385000

CASSCF C29 Triplet

6	1.780422783	5.140970690	-0.154394204
6	0.612299494	5.391780826	-0.157971809
6	-3.230014241	4.741830554	-0.156959734
6	-4.245260224	4.111144354	-0.152595161
6	-6.271631270	0.804761348	-0.120780541
6	-6.379394832	-0.385627483	-0.107328226
6	-6.157479424	-1.746127099	-0.091060592
6	-5.676519419	-2.840345309	-0.077350578
6	-4.860842812	-3.951087047	-0.063512856
6	-1.542874378	-5.611565396	-0.040892683
6	-0.167441120	-5.687281693	-0.037917729
6	3.350598804	-4.449213475	-0.042325427
6	4.422996719	-3.582914890	-0.047946630
6	5.210973301	-2.685572124	-0.054644031
6	5.980228815	-1.538963729	-0.064854704
6	6.266904393	-0.332389715	-0.079945921
6	6.511843852	0.984522006	-0.097250818
6	5.853024959	2.151425475	-0.114692194
6	5.189234429	3.198392424	-0.130544511
6	-5.851167313	2.117063179	-0.134699411
6	-5.203549614	3.121698026	-0.144245297
6	-3.927909504	-4.698380896	-0.054074756
6	-2.705278019	-5.333252956	-0.045502559
6	-1.946948024	5.242694259	-0.159887333
6	-0.764907217	5.418482002	-0.159961035
6	1.011522616	-5.491499279	-0.037649589
6	2.314598375	-5.044401747	-0.039136555
6	3.067724332	4.648087239	-0.148341936
6	4.097415367	4.043276945	-0.141101059

DFT/BHLYP C29 Triplet

6	1.902645000	5.576532000	0.003356000
6	0.705063000	5.860075000	0.005118000
6	-3.025200000	5.046993000	-0.000338000
6	-4.001101000	4.325436000	-0.003371000
6	-5.822457000	0.952401000	-0.007832000
6	-5.897695000	-0.252686000	-0.005872000
6	-5.691011000	-1.581826000	-0.002097000
6	-5.246313000	-2.706019000	0.001456000
6	-4.503117000	-3.822759000	0.005985000
6	-1.325454000	-5.739445000	0.007321000
6	-0.010726000	-5.899865000	0.006326000
6	3.520502000	-4.710441000	0.000637000
6	4.478898000	-3.833831000	-0.001395000
6	5.174003000	-2.788906000	-0.001905000
6	5.672739000	-1.605328000	-0.003195000
6	5.868667000	-0.351372000	-0.003391000

6	5.827978000	0.917676000	-0.004169000
6	5.477946000	2.152846000	-0.003318000
6	4.915209000	3.275162000	-0.002698000
6	-5.453080000	2.245723000	-0.008024000
6	-4.873678000	3.306906000	-0.006720000
6	-3.620307000	-4.655367000	0.007492000
6	-2.484123000	-5.355093000	0.009380000
6	-1.814878000	5.607937000	0.003202000
6	-0.618951000	5.854211000	0.004422000
6	1.211526000	-5.759009000	0.003692000
6	2.461612000	-5.359333000	0.001524000
6	3.096234000	5.032061000	0.001778000
6	4.070071000	4.260696000	-0.001016000
